# Evolution of the calcium feedback steps of vertebrate phototransduction

**DOI:** 10.1098/rsob.180119

**Published:** 2018-09-26

**Authors:** Trevor D. Lamb, David M. Hunt

**Affiliations:** 1 Eccles Institute of Neuroscience, John Curtin School of Medical Research, The Australian National University, Australian Capital Territory 2600, Australia; 2 Centre for Ophthalmology and Visual Science, The Lions Eye Institute, The University of Western Australia, Western Australia 6009, Australia; 3 School of Biological Sciences, The University of Western Australia, Western Australia 6009, Australia

**Keywords:** evolution, phototransduction, recoverin, guanylyl cyclase activating protein, guanylyl cyclase, sodium/calcium–potassium exchanger

## Abstract

We examined the genes encoding the proteins that mediate the Ca-feedback regulatory system in vertebrate rod and cone phototransduction. These proteins comprise four families: recoverin/visinin, the guanylyl cyclase activating proteins (GCAPs), the guanylyl cyclases (GCs) and the sodium/calcium-potassium exchangers (NCKXs). We identified a paralogon containing at least 36 phototransduction genes from at least fourteen families, including all four of the families involved in the Ca-feedback loop (recoverin/visinin, GCAPs, GCs and NCKXs). By combining analyses of gene synteny with analyses of the molecular phylogeny for each of these four families of genes for Ca-feedback regulation, we have established the likely pattern of gene duplications and losses underlying the expansion of isoforms, both before and during the two rounds of whole-genome duplication (2R WGD) that occurred in early vertebrate evolution. Furthermore, by combining our results with earlier evidence on the timing of duplication of the visual G-protein receptor kinase genes, we propose that specialization of proto-vertebrate photoreceptor cells for operation at high and low light intensities preceded the emergence of rhodopsin, which occurred during 2R WGD.

## 1. Background

The rod and cone photoreceptors of the vertebrate duplex retina, used, respectively, for night and day vision, employ distinct protein isoforms for many of the components of the transduction cascade. These cells therefore represent a unique evolutionary system, where the same process (detection of light) uses a distinct set of genes in different classes of cell. It has been established that a major factor leading to the emergence of this duplex system was the occurrence of the two rounds of whole-genome duplication (2R WGD) [1] that are known to have occurred at a very early stage in the evolution of vertebrates. A series of studies from Larhammar's group [2–7] reported evidence for extensive expansion of phototransduction gene isoforms during 2R WGD. Recently, we extended those investigations by including genes from several basal vertebrate lineages (the jawless or agnathan vertebrates, cartilaginous fish and basal bony fish). We obtained eye transcriptomes for nine taxa of interest, and applied molecular phylogenetic analysis to curated sets of gene families. First, we examined the gene families encoding the proteins involved in *activation* of the light response (the opsins; transducins, GNATs; phosphodiesterases, PDE6s; cyclic nucleotide-gated channels, CNGCs) [8,9]. Then we examined the genes involved in *shut-off* of the light response (the G-protein receptor kinases, GRKs; arrestins; regulators of G-protein signalling, RGS9s; G-protein *β* subunit 5, G*β*5; RGS anchor proteins, R9APs) [10]. We confirmed the importance of 2R WGD in establishing distinct rod/cone isoforms in both the activation and the shut-off steps in the phototransduction cascade, and we presented likely scenarios for the gene duplications and losses that occurred during the evolution of each of the 10 gene families that we examined.

Here, we analyse the four gene families encoding the proteins that contribute to the important Ca-feedback regulatory system that underlies photoreceptor light adaptation and that acts as a protective mechanism against excessive concentrations of either cyclic GMP (cGMP) or cytoplasmic Ca^2+^; the corresponding protein families are: recoverin/visinin, the guanylyl cyclase activating proteins (GCAPs), the guanylyl cyclases (GCs) and the sodium/calcium–potassium exchangers (NCKXs). In the Results section, we begin by identifying two paralogous regions: one that includes 28 phototransduction genes, and a second that includes another seven phototransduction genes. Thereafter we consider in turn the four gene families that contribute to Ca feedback. In each case, we examine gene synteny and then construct molecular phylogenies. Together, these analyses of synteny and phylogeny enable us to determine the likely scenario of gene duplications that gave rise to the vertebrate photoreceptor's Ca-feedback regulatory system.

Throughout this paper, we have chosen to employ the protein name when referring to the encoding gene, for several reasons. First, for a number of the proteins, the genes have been lost in human and the orthologues in other taxa do not have accepted gene names. Second, for some genes there is inconsistency across vertebrates in the naming of orthologues (e.g. *GUCY2D* and *Gucy2e* for the same orthologue). And finally, we think that the protein names are more easily understood among photoreceptor neuroscientists.

### 1.1. Recoverin and visinin

Recoverin, visinin and the GCAPs are members of a family of neuronal calcium sensor proteins (NCSs; reviewed in [11–13]) characterized by a sequence of roughly 200 residues with a highly conserved secondary structure comprising four EF-hand motifs. In comparison with other NCS proteins, the first EF-hand in these photoreceptor proteins has an altered amino acid sequence that prevents it from binding Ca^2+^; for the GCAPs, the remaining three motifs (EF2–EF4) bind Ca^2+^, whereas only EF2 and EF3 bind Ca^2+^ in recoverin and visinin. Functionally, the GCAPs exert a powerful dependence of guanylyl cyclase activity on Ca^2+^ concentration, whereas recoverin provides only a weak Ca dependence of phosphorylation activity by the G-protein receptor kinases (GRKs).

Visinin was purified from chicken retina and identified as a Ca-binding protein in cones in 1983 [14] and was cloned in 1990 [15]. The following year, two closely related proteins were discovered: S-modulin in frog rods [16], and recoverin in mammalian rods [17], though the latter was incorrectly reported to be the Ca-sensitive regulator of guanylyl cyclase activity. Since then a degree of misunderstanding has surrounded the three proteins, in part because mammals lack the gene for visinin, and also because a distinct mammalian gene has been named ‘visinin-like’ (*VSNL1*) and sometimes confused with visinin. Our analysis will show that recoverin has been lost from sauropsids (reptiles and birds), whereas visinin has been lost from cartilaginous fish and mammals (and possibly from coelacanth). As a result, it is only in amphibia and bony fish that both isoforms can readily be found. Our analysis will also show that recoverin and S-modulin are the same.

The cellular and molecular mechanism whereby recoverin exerts a degree of Ca sensitivity on the recovery phase of the photoresponse is partly understood (reviewed in [12]). Recoverin is myristoylated at its N-terminus, and at low Ca^2+^ concentrations (as occur in bright light), the myristoyl group is buried in a hydrophobic pocket within the protein [18,19]; in this state recoverin interacts only weakly with membranes, and has little effect. But when Ca^2+^ binds at the higher Ca^2+^ concentration characteristic of the dark state, a conformational change occurs (a ‘myristoyl switch’ [20]) involving exposure of both the myristoyl group and the hydrophobic pocket. This enables recoverin to bind to the membrane and to interact with GRK1, inhibiting it and thereby slowing the phosphorylation of activated rhodopsin. As a result, the elevated Ca^2+^ level at low light levels leads to an increase in lifetime (and hence increased effectiveness) of any rhodopsins that are activated. Conversely, in bright light the lifetime of activated rhodopsin is shortened. The light responses of recoverin knockout mice are however only moderately shorter than those of WT mice [21], indicating that recoverin's role is modest. Unlike in the case for GCAPs, the binding of Mg^2+^ appears to have little effect on recoverin [18,19].

In contrast to this relatively subtle effect of recoverin in rods, visinin in cones may have a more pronounced effect. *In vitro* experiments on frog retina have shown that visinin and recoverin exert indistinguishable effects on the GRKs [22]. However, the expression level of visinin in frog cones is 20-fold higher than the expression level of recoverin in rods [22], providing the potential for a more profound role of visinin *in vivo*.

### 1.2. Guanylyl cyclase activating proteins, GCAPs

Within the extensive set of neuronal calcium sensor proteins, the vertebrate genome includes a family of guanylyl cyclase regulatory proteins (reviewed in [11–13]), comprising several ‘activating’ proteins (GCAPs) and a single so-called ‘inhibitory’ protein (GCIP). Our analyses of synteny and phylogeny will divide GCAPs into six sub-families, with teleost fish possessing 3R duplicates of several of these [23–25]. The best-studied members are GCAP1 (encoded by *GUCA1A*) and GCAP2 (encoded by *GUCA1B*); these two genes are arranged tail-to-tail in virtually all tetrapods, as well as in spotted gar, though not in teleosts.

In mammalian cones, the predominant isoform is GCAP1 [26]; the level of GCAP2 is species-dependent, but is always much lower than GCAP1, or even absent. In zebrafish, an isoform that we identify here as one member of the pair of GCAP1 duplicates (zGCAP5, here referred to as GCAP1b) is cone-specific [23,24]. In mammalian rods, GCAP1 and GCAP2 are co-expressed, with the level of GCAP2 being higher [27].

A third isoform, GCAP3 (encoded by *GUCA1C*), occurs in many species, and is expressed only in cones, at least in human and zebrafish [28]; in the latter species, both 3R duplicates (zGCAP3 and zGCAP4, here referred to as GCAP3a and GCAP3b) are cone-specific [24,28,29]. A fourth isoform, GCAP1-L, closely similar to GCAP1 and GCAP3, is often overlooked, probably because it has been lost from mammals. Finally, another pair of isoforms closely similar to GCAP2, and here referred to as GCAP2-A and GCAP2-B, occur in a number of vertebrate taxa. As far as we are aware, very little is known about these last three isoforms (GCAP1-L, GCAP2-A and GCAP2-B), except that in zebrafish the only 3R duplicate of GCAP2-B (zGCAP7) is cone-specific [25].

GCAPs provide very powerful Ca-sensitive activation of guanylyl cyclases (GCs) [30]. Each EF-hand, apart from the first, has the capacity to bind either Ca^2+^ or Mg^2+^; the first EF-hand is instead modified to interface with GCs [31]. The molecular mechanism of GCAP activation at lowered Ca^2+^ concentrations involves the binding of Mg^2+^ [32] to EF-2 and EF-3, thereby inducing a conformational change; the role of EF4 is unclear. In contrast to the case for recoverin, the overall change in tertiary structure for GCAP1 is relatively small [33]; in particular, the myristoyl group in GCAP1 remains fully buried in both states [34–36]. GCAP2 is also myristoylated, but whereas the removal of the myristoyl group results in a sevenfold reduction in activity of GCAP1, it has little effect on GCAP2 [37].

Recent evidence has shown that GCAP1 forms a functional homodimer [33], suggesting a 2 : 2 stoichiometry of interaction with the GC homodimer. *In vitro* experiments with mammalian proteins have shown that GCAP1 and GCAP2 are able to activate the two GCs, GC-E and GC-F, with comparable efficacy. Furthermore, mammalian rods co-express both GCs, as well as both GCAPs. A clear functional specialization has been established in rods *in vivo*, with GCAP1 primarily regulating GC-E (Ret-GC1) [38]. However, GCAP1 and GCAP2 appear to compete for overlapping site(s), and so the role of GCAP2 remains somewhat enigmatic [38–40]. Functionally, the Ca^2+^ sensitivity of a cell's cyclase activity is determined by its GCAP(s), and GCAP1 activates at a higher Ca^2+^ concentration than GCAP2 (approx. 140 nM cf. approx. 50 nM). For rods, dim illumination causes only a moderate decline in Ca^2+^ concentration and therefore triggers GCAP1/GC-E activation alone, whereas bright illumination lowers the Ca^2+^ concentration substantially and hence additionally triggers GCAP2/GC-F activation [39].

GCIP has been termed ‘inhibitory’ [41], but this nomenclature is potentially misleading. On its own, GCIP stimulates GC only very slightly, and in the presence of a constitutively active GCAP, it has no effect on GC activity when the Ca^2+^ concentration is very low [41]. On the other hand, at very high Ca^2+^ concentrations the activation of GC by the constitutively active GCAP is blocked [41], presumably as a result of competition for binding to the GC. However, it has not been established that GCIP plays an inhibitory functional role *in vivo*. Finally, although GCIP was reported to label cone photoreceptors strongly in the inner segment and synaptic terminal, but not in the outer segment, examination of the immunofluorescence images (fig. 6A of [41]) suggests that it may also be present in the outer segment.

### 1.3. Guanylyl cyclases, GCs

We will adopt the protein names GC-A to GC-G assigned by IUPHAR/BPS for the seven transmembrane guanylyl cyclases encoded by the mammalian genome (www.guidetopharmacology.org/GRAC/FamilyDisplayForward?familyId=662). The properties of these GCs have recently been reviewed in [42]. The two mammalian photoreceptor isoforms are GC-F (=Ret-GC2), encoded by *GUCY2F*, and GC-E (=Ret-GC1), encoded by *Gucy2e* in mouse and a number of other mammals, though by *GUCY2D* in human and many other species. A third isoform, GC-D, often referred to as the ‘olfactory’ GC, does not occur in primates but is present in most vertebrate taxa; in mouse, the encoding gene is named *Gucy2d*, but in many taxa it is unnamed. In zebrafish, our phylogenetic results identify the gene names as follows: *gc3* = GC-E (Ret-GC1), *gc2* = GC-F (Ret-GC2), *gucy2f* = GC-D (olfactory); in the Discussion we will consider other reported claims of gene orthology. In any case, there is considerable potential for confusion regarding the isoform being referred to when using the gene names, and therefore (as noted above) we will use the protein names. We will show that the third so-called ‘olfactory’ isoform is expressed in the retina, at least in many aquatic taxa, including bony, cartilaginous and agnathan fish. Indeed, in our retina transcriptomes for bowfin and Florida gar, the transcript levels for GC-D are similar to those for GC-F, and considerably higher than for GC-E (see §2.5.3).

Several studies have shown that GC-E and GC-F are co-expressed in the rods of jawed vertebrates. In mouse rods, GC-E is present at approximately 5× higher concentration than GC-F [39], yet the catalytic activity of GC-F exceeds that of GC-E, with the result that GC-F contributes around 25–30% of the maximal cyclase activity. In cones of WT mouse [43] and zebrafish [24], it has been reported that the only GC present is GC-E. In humans, mutations in the GC-E gene (*GUCY2D*) are known to cause Leber congenital amaurosis type 1 (LCA1) [44], a recessive childhood disease associated with severe vision loss, and dominant cone-rod dystrophy [45]. On the other hand, no human retinal diseases have yet been linked to mutations in the GC-F gene (*GUCY2F*).

These photoreceptor GCs synthesize cGMP, at a rate set by the cytoplasmic Ca^2+^ concentration via the extent of their activation by GCAPs; however, the molecular mechanism of activation by GCAPs has not yet been elucidated. The cyclase molecule is a long membrane-spanning homodimer, in which seven functional domains have been identified [40,46,47]. In rods, the so-called extracellular domain is in fact intradiscal; of the remaining domains we will mention only two. Generation of a functional catalytic site requires dimer formation, and this is achieved through subunit interaction via the α-helical coiled-coil motif of a dimerization domain (DD) in both partners [48]. In the dimer, the paired cyclase catalytic domains (CCDs) form the catalytic centre where cGMP is synthesized [49]. Finally, it is interesting to note that during their synthesis and transport to the outer segment, GCs appear to be protected from activation by the binding of a Ca-insensitive protein, RD3 [43,50].

### 1.4. Sodium/calcium-potassium exchangers, NCKXs

Ca^2+^ ions are extruded from rod and cone outer segments by a sodium/calcium–potassium exchanger, NCKX (reviewed in [51,52]), that is able to operate at very low cytoplasmic Ca^2+^ levels because it uses both the inward concentration gradient of Na^+^ and the outward concentration gradient of K^+^. The exchanger operates ‘electrogenically’ [53], with a net influx of one positive charge per Ca^2+^ extruded, because each cycle has a stoichiometry of four Na^+^ ions transported inward, in exchange for one Ca^2+^ ion plus one K^+^ ion (i.e. three positive charges) transported outward [54–56]. As a result, the operation of this exchanger can be measured in intact cells under suitable conditions, by recording the electrogenic current.

In darkness, when CNGCs are held open by a moderate level of cGMP, there is a steady influx of Ca^2+^ ions through the relatively non-selective channels, and this influx is balanced by an equal efflux of Ca^2+^ driven by the NCKX, generating a moderately high free Ca^2+^ concentration of 200–500 nM [56–58]. In bright light, all the CNGCs are closed so that the influx of Ca^2+^ stops, but initially the efflux continues, resulting in a drop in cytoplasmic Ca^2+^ concentration [59]. This drop is crucial in triggering rapid recovery of the electrical response and in mediating light adaptation [60,61].

In the rod outer segment, the NCKX protein forms a tight 2 : 1 association with CNGCs, with one NCKX binding to each of the two *α*-subunits of the CNGC [62,63]. This protein complex in the plasma membrane additionally interacts with peripherin-2 in the rim of the disc membranes via the glutamic acid-rich protein component of the CNGC β-subunit [64], thereby apparently providing stabilization of the outer segment disc structure.

Rods express NCKX1 (encoded by *SLC24A1*) while cones express NCKX2 (encoded by *SLC24A2*), and we will present evidence that these two isoforms arose during 2R WGD. They are members of what has been designated Group 2 of a super-family of Ca^2+^/cation antiporters (reviewed in [65]). Group 1 includes NCKX3, NCKX4 and NCKX5, and is likewise found only in animals; Group 3 is found in land plants and algae.

## 2. Results

### 2.1. Syntenic arrangement of phototransduction genes

#### 2.1.1. Many phototransduction genes are located in a paralogous region

**Figure 1.**
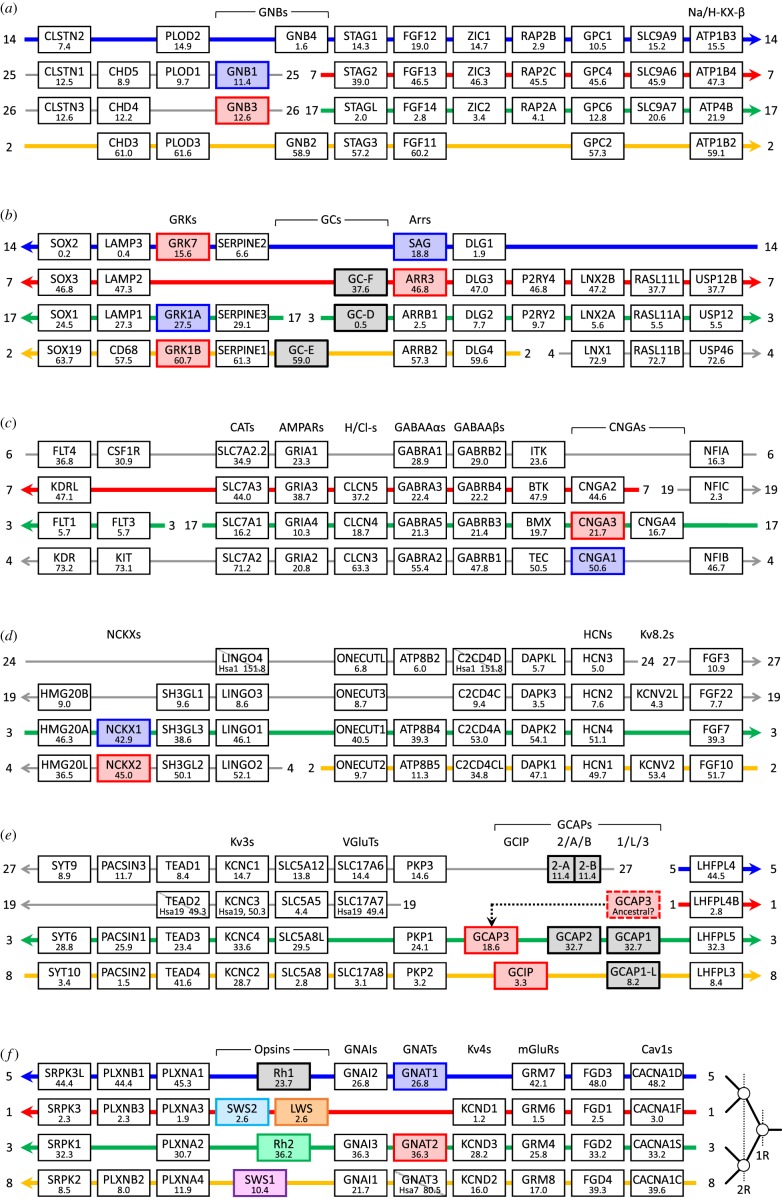
Syntenic arrangement of 62 families of genes located in the neighbourhood of phototransduction genes. The 28 genes involved in phototransduction (including nine that participate in Ca-feedback regulation) are shown either coloured or shaded. Red indicates preferential expression in cones; blue, preferential expression in rods; grey, expression in rods and cones; for the visual opsins, the colours instead provide an indication of spectral sensitivity. The rows represent spotted gar linkage groups (chromosomes) and the adjacent numbers identify the individual linkage groups; thus, ‘14’ indicates LG14. The diagram has arbitrarily been divided into six panels (*a–f*), and where a linkage group continues across a break between panels this is indicated by an arrow at the end of one panel and at the start of the next. The number below each gene identifier gives the gene location on the spotted gar linkage group in Mb. The order of gene families is arbitrary, although as far as possible we have arranged them in locally increasing or decreasing order of gene position in Mb on LG3/LG17 (green row). The diagram attempts to provide a coherent picture of the likely continuity of the four paralogous chromosomal regions in the ancestral post-2R genome. However, there is inevitable uncertainty at each break in linkage group coverage. To address this, our illustrated arrangement additionally takes into account the chromosomal locations of genes in human and chicken, as tabulated in electronic supplementary material, table S1. For those regions where we feel reasonably confident of the continuity of each postulated ancestral chromosome we have used thicker coloured lines; for regions where we are less confident the lines are thinner and grey. Genes with a diagonal strike-through are missing from the spotted gar genome, and their presumed locations have been derived from human and/or chicken. In panel (*e*), the dotted arrow links the postulated ancestral location of GCAP3 (*GUCA1C*) to its current location in spotted gar (see Text). The branching patterns sketched at the bottom right represents the order of 1R and 2R duplications deduced recently for the GRKs and arrestins [10], and for the GNAIs/GNATs [9].

In searching for synteny among the genes encoding the proteins of the calcium feedback loop, we were struck by the close proximity of many sets of phototransduction genes to each other. Figure 1 shows the configuration of more than 200 genes from 63 gene families for spotted gar (*Lepisosteus oculatus*), with the linkage groups (chromosomes) indicated by horizontal lines; for pictorial convenience the diagram has been split into six sections (*a–f*). To our astonishment, we found that nine families comprising 28 genes directly involved (or implicated) in phototransduction were located close to each other. The occurrence of multiple families of genes, with paralogues arranged in a closely similar sequence across four chromosomes, is a signature of the remnants of a quartet (paralogon) that arose during the two rounds of whole-genome duplication (2R WGD) that occurred very early in vertebrate evolution [1]. Although we are confident that such a quartet arrangement holds at local levels within figure 1, we cannot be sure that the indicated arrangement of rows holds globally, across the entire set of genes.

The rationale for our choices of row continuity in figure 1, at ‘breaks’ in chromosomal arrangement, is explained in the Methods (§4.1) and is based on comparison of gene locations across three taxa (spotted gar, human and chicken) as tabulated in electronic supplementary material, table S1. For the regions where the horizontal lines are shown thicker and coloured, we think it very likely that the ancestral arrangement remains continuous across each of the four colours. On the one hand, for the stretches of figure 1 shown with thinner grey lines, we are less certain, and it is possible that some chromosomal regions may need to be swapped. For example, in panel (*c*), it may be that LG6 and LG4 should be interchanged between rows 1 and 4; likewise, in panels (*d,e*), it may be that LG19 and LG24/LG27 should be interchanged. On the other hand, our subsequent phylogenetic analysis for the NCKX sequences (§2.6) provides circumstantial evidence to suggest that in panel (*d*) the third and fourth rows (containing *SLC24A1* and *SLC24A2*) are correctly paired.

Overall, we are confident that panel (*b*) forms a paralogon, and likewise that panel (*f*) also forms a paralogon, and furthermore we think it likely that these two regions actually comprise two regions of the same paralogon. Although we acknowledge uncertainty about continuity of the indicated rows (e.g. across the middle panels, *c–e*), we hypothesize that the entire diagram may represent a single paralogon.

In figure 1, there are five instances where we have represented a gene family using more than one column (horizontal braces), on the basis of evidence for the occurrence of duplication(s) that preceded 2R WGD. We have previously reported two of these cases: for the CNGAs [8] and the visual opsins [9]. In the phylogenetic analyses in subsequent sections, we provide corresponding evidence in the case of the GCAPs (§2.4) and the guanylyl cyclases (§2.5). Finally, we note that in figure 1*e*, the location of GCAP3 (encoded by *GUCA1C*) is indicated as having been translocated in the spotted gar genome; the position of this gene also appears unusual in several other genomes (electronic supplementary material, table S2) and will be considered in more detail in §2.4.

#### 2.1.2. A second paralogous region containing phototransduction genes

Inspection of figure 1 shows the absence of a few sets of phototransduction genes (e.g. for the PDE6 catalytic and regulatory subunits, the G-protein γ-subunits, and recoverin and visinin). In attempting to identify gene synteny for recoverin and visinin, a significant complication is that relatively few taxa retain the genes for both isoforms; notably, though, ray-finned fish and amphibia frequently retain both. The genome that we have primarily analysed is that of the spotted gar, but unfortunately the current assembly does not include the gene for visinin; we suspect that the gene exists in this species, because our retinal transcriptome for the closely related Florida gar (*L. platyrhincus*) contains transcripts for both recoverin and visinin. Therefore, we also examined the genomes of *Xenopus tropicalis* and *Anolis carolinensis*, though these have the disadvantage of shorter scaffold lengths. In addition, and despite the complication of 3R, we chose to examine the zebrafish genome, which contains a pair of recoverin genes (*RCVRNA* and *RCVRNB*) and a pair of visinin genes (named *RCVRN2* and *RCVRN3*); to minimize confusion in relation to these isoforms, we will refer to the latter pair as visinin-A and visinin-B.

**Figure 2.**
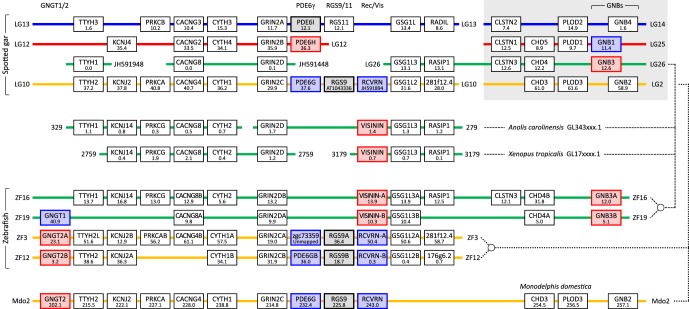
Syntenic arrangement of genes neighbouring recoverin and visinin. Top four rows are spotted gar linkage groups that appear to form a 2R paralogon. The genes at the top right (grey background) also appear in figure 1, and this provides the basis for colouring the top row blue and the fourth row orange; the second and third rows have been coloured red and green, respectively, for reasons explained in the text, but this identification is not secure. As the genome assembly for spotted gar does not contain visinin, we also included genes from the unplaced scaffolds GL343279.1 and GL343329.1 of green anole, and GL173179.1 and GL172759.1 of *Xenopus*; these are shown as the next two rows. For spotted gar, five of the illustrated genes are on unplaced scaffolds; *RCVRN* and *RGS9* have been placed on LG10/LG2 by comparison with mammalian genomes (e.g. opossum, in bottom row), *TTYH1*, *CACNG8* and *GRIN3D* have been placed on the same row as LG26 by comparison with anole. Next four rows are for zebrafish, and show genes on chromosomes ZF3/ZF12 and ZF16/ZF19, that include the 3R copies of recoverin and visinin. Bottom row is for opossum chromosome 2, and is presented as evidence supporting continuity of the orange row LG10/LG2 for spotted gar. Bold outlines denote phototransduction genes, with colour coding as in figure 1. Dashed and dotted lines link the rows for visinin and recoverin, respectively; open circles denote 3R duplications in zebrafish. Note that the genes we designate as *VISININ-A* and *VISININ-B* are named *RCVRN2* and *RCVRN3* in Ensembl and NCBI; we have also made some minor changes to a few other gene names to aid comparison across the three species and for the avoidance of confusion. Gene locations are from Ensembl Release 93. The locations of *PDE6H* and *PDE6I* in spotted gar have been obtained from [7].

Figure 2 provides what we consider to be compelling evidence for a paralogon containing five phototransduction gene families, namely: recoverin/visinin, *GNB1/3*, *PDE6G/H/I*, *RGS9*/*11* and *GNGT1*/*2*. The top four rows are from spotted gar, and provide clear evidence for the existence of a paralogon; the upper right section (grey background) shows continuity with figure 1. Although several anticipated genes are absent from LG26, we found orthologues in the anole and *Xenopus* genomes (next two rows), providing evidence that permitted us to identify the location of the visinin gene. Additional confirmation for the structure of this paralogon is provided by the subsequent four rows, which examine the chromosomal regions in zebrafish that contain paralogues of recoverin and visinin. (To avoid excessive complication, we have *not* presented data for these other three taxa corresponding to the top two spotted gar linkage groups, LG14/LG13 and LG25/LG12.) Finally, to provide evidence for contiguity across the extent of figure 2, the bottom row shows the genes from a single section on chromosome 2 of the opossum, *Monodelphis domestica*.

We interpret the orange rows (that are joined by the dotted lines) to identify the common arrangement of genes in the vicinity of recoverin. Thus, zebrafish chromosomes ZF3 and ZF12 show the 3R duplicates from a presumed ancestral post-2R chromosome containing recoverin, which is now represented in spotted gar by LG10/LG2 and in opossum by chromosome 2. Likewise, we interpret the green rows (that are joined by the dashed lines) to identify the genes in the vicinity of visinin. Thus, zebrafish chromosomes ZF16 and ZF19 show the 3R duplicates from a presumed ancestral chromosome containing visinin, which existed after 2R and is now represented in spotted gar by LG26 and in anole and frog by several short scaffolds; various of those ancestral genes have subsequently been lost from spotted gar, anole and frog.

By comparison with the human and chicken genomes (data not shown), and also with the opossum genome (bottom row), it is clear that the top row and the fourth row in figure 2 represent the corresponding top and fourth rows in figure 1, and accordingly we have coloured them blue and orange, respectively. From these results, we conclude that the expansion of recoverin and visinin occurred during 2R WGD. However, despite extensive efforts, we were not able to conclusively associate the second or third rows in figure 2 with individual rows in figure 1. We have chosen to place LG26 on the third row, and therefore to colour it green, because our subsequent phylogenetic analysis (§2.3.1) shows a marginal preference for the divergence of visinin and recoverin having occurred at the second round of 2R WGD. However, we cannot rule out the possibility that LG26 should actually be placed on the second row, so that the red and green colouring of rows would be interchanged throughout the figure. Indeed, we gained the impression that the relationship between these rows may not be consistent across taxa.

By combining the results from figures 1 and 2, we conclude that almost all of the genes directly involved in phototransduction (at least 35 genes, for activation, shut-off and Ca feedback) appear to be located in a single paralogon. The most important gene family that is not present in figures 1 or 2 is *PDE6A*/*B*/*C*. Recently, Lagman *et al.* [7] showed that this set of genes resides in a paralogous region, and although in spotted gar the genes are located on LG6, LG2 and LG5 (which appear prominently in figure 1), we have not found conclusive evidence linking them to the paralogon of figures 1 and 2. In the Discussion, we will give further consideration to this and other potential members of the paralogon.

### 2.2. Sequences obtained from transcriptomes for agnathans and basal jawed vertebrates

We searched our transcriptome data (as described in Methods, §4.2) for sequences that were close hits against each of the four families of Ca-feedback components: recoverin and visinin, GCAPs, guanylyl cyclases and NCKXs. For the sequences obtained, we conducted multiple sequence alignment against a curated set of database sequences, followed by phylogenetic analysis and structural analysis, and we used the combined analysis in annotating the sequences. The 73 new sequences have been deposited in GenBank with nucleotide accession numbers MH577347-MH577419. Features of the entire set of sequences are listed in electronic supplementary material, table S3. For ease of reference, we now summarize in table 1 the transcript levels for isoforms from selected species; these cases will be referred in the following sections.

**Table 1.** Transcript levels in selected species. Numbers represent transcript levels in RPKM-CDS (i.e. calculated over the coding region), and have been taken from electronic supplementary material, table S3. Entries are intentionally empty for agnathan-specific isoforms in columns for gnathostomes, and likewise for gnathostome-specific isoforms in columns for lampreys. A dash indicates transcripts not detected or, for mammals, not expressed in this class of photoreceptor. ✗indicates gene lost from mammals. ✓✓ indicates isoform generally expressed in this class of mammalian photoreceptor. ✓ indicates isoform expressed either at low level, or only in some cells of this class. ? indicates uncertain expression.

	pouched lamprey, *Geotria australis*	short-headed lamprey, *Mordacia mordax*	bluespot ray, *Neotrygon kuhlii*	reef shark, *Carcharhinus amblyrhincos*	bowfin, *Amia calva*	Florida gar, *Lepisosteus oculatus*	mammal, cones	mammal, rods
RecVis-X	1640	434						
RecVis-Y	35	68						
visinin			—	—	904	384	✗	
recoverin			2163	—	1701	1261	?	✓✓
GCAP1-X	252	86						
GCAP1-Y	6	—						
GCAP1-L			310	461	337	239	✗	
GCAP1			—	—	177	273	✓✓	✓
GCAP3			—	—	57	41	✓	—
GCAP2	299	181	1472	1507	420	117	✓	✓✓
GCAP2-A			—	—	71	182	✗	
GCAP2-B			—	—	36	47	✗	
GCIP	—	—	—	—	—	—	✗	
GC-X	11	22						
GC-E			33	—	5	—	✓✓	✓
GC-D			47	63	18	20	—	—
GC-F			88	102	—	21	✓	✓✓
NCKX-X	1	—						
NCKX-Y	1	—						
NCKX2	15	20	12	3	22	8	✓✓	—
NCKX1			86	199	22	5	—	✓✓

### 2.3. Recoverin and visinin

#### 2.3.1. Molecular phylogeny of recoverin and visinin

For recoverin/visinin, we considered gene synteny in §2.1.2, and so we now move directly to phylogeny. By BLASTing against chicken visinin, we located more than a dozen very close hits across reptiles, birds and bony fish, though most of these sequences were named recoverin, recoverin-like, recoverin 2 or visinin-like. In addition, our eye transcriptomes provided transcripts for a visinin, expressed at high level, in bowfin and Florida gar. For agnathan species, we found a pair of hits to recoverin/visinin in *P. marinus* and *L. camtshaticum*, and our transcriptome provided corresponding pairs in both our lamprey species, but none from hagfish. The multiple sequence alignment for our curated set of recoverins and visinins is presented as electronic supplementary material, file S1.

For the set of recoverins and visinins, from jawed and agnathan vertebrates, the sequence alignment over the first 189 residues was so tight that there were no gaps at all, while for the outgroup members, there were just two gaps (of a single residue and a pair of residues). However, for the remaining dozen or so C-terminal residues, MAFFT and Clustal generated somewhat different alignments. Therefore, in constructing phylogenies, we tried both alignments as well as truncating all sequences after residue 189; in addition, we tested the WAG and LG substitution models. In all cases, the resulting trees were closely similar.

We encountered a minor problem rooting the trees, because the nearest outgroup sequences we could locate were quite distant, with an average of approximately 0.7 substitutions per residue. Therefore, we began by ignoring the outgroup and constructing an unrooted phylogeny. The tree we obtained is presented in electronic supplementary material, figure S1, and had identical topology (Robinson–Foulds distance = 0) with the WAG and LG substitution models. Importantly, support for the four vertebrate clades is unanimous (in terms of IQ-Tree's approximate bootstrap percentage, hereinafter referred to simply as bootstrap support). The four branches are long, and the two jawed vertebrate clades are separated from the two agnathan clades by only a short branch, though support for this topology is quite high (95% for both WAG and LG). With four clades, there are two other possible topologies, in each case with a jawed vertebrate clade sister to an agnathan vertebrate clade. When we applied tests of topology, we found that neither of those alternative topologies could be rejected in any of the three tests. Therefore, we conclude that the four vertebrate clades are genuinely distinct from each other, but we cannot be sure of their topology relative to each other.

**Figure 3.**
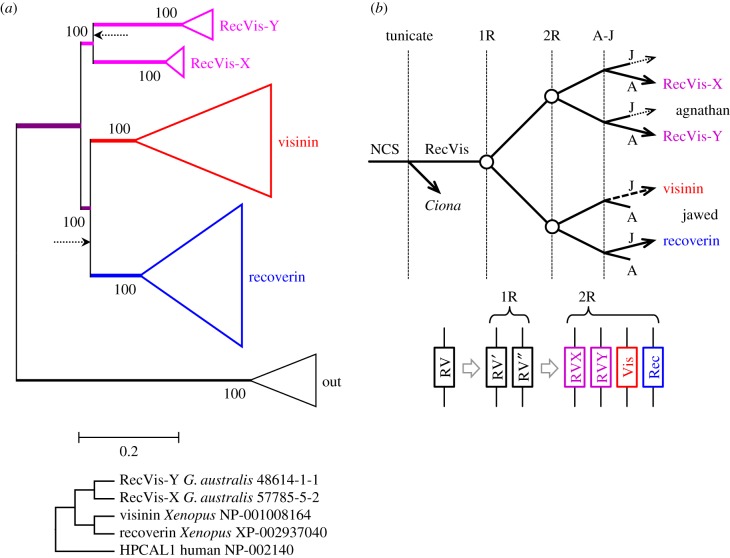
Molecular phylogeny and proposed gene duplications and losses for recoverin and visinin. (*a*) Constrained molecular phylogeny for recoverin and visinin sequences from jawed and agnathan vertebrates, in collapsed form. The fully expanded tree is shown in electronic supplementary material, figure S2; identical topology was obtained using the WAG and LG substitution models. The two dotted arrows show the positions obtained for the root of the unconstrained tree, with the WAG model (lower arrow) and the LG model (upper arrow). The constraint tree that was applied is shown below the main panel; by constraining just two jawed vertebrate sequences and two agnathan sequences, the support level became unanimous for each of the five sub-trees. (*b*) Proposed scenario for gene duplications and losses. The only losses are presumed to have occurred following agnathan-jawed (*a–j*) speciation. Although the pattern with visinin shown with a dashed arrow has the highest level of support, we could not rule out the possibility that visinin is instead sister to one or other of the two agnathan sequences, as shown by the dotted arrows (see text).

When we included the outgroup sequences in the analysis, the root in the unconstrained tree was placed either adjacent to the recoverins (WAG model) or adjacent to the agnathan RecVis-Y clade (LG model). These alternative positions obtained for the root in the unconstrained trees are sketched by the dotted arrows in the constrained tree that we present in figure 3*a*. The discrepancy between the root positions obtained for the two substitutions models suggests that the large phylogenetic distance to the outgroup sequences precludes reliable placement of the root in an unconstrained phylogeny.

We next examined the consequences of constraining the topology to be consistent with divergence during 2R, as required to explain our gene synteny results in figure 2. The simplest way of achieving this, and the one that turned out to exhibit the highest likelihood, was to shift the root by one node (for either the WAG or LG substitution model), to the position shown in figure 3*a*; the fully expanded tree is presented in electronic supplementary material, figure S2. Interestingly, despite the different root positions in the two unconstrained trees, the constrained trees had identical topology for the two substitution model. Application of the constraint caused only a small change in log likelihood (ΔlogL) of 4.3 with WAG, or 4.2 with LG, and in both cases the constrained tree passed all three tests of topology when compared with the corresponding unconstrained tree, with a probability in the approximately unbiased test of *p*-AU = 0.47 for WAG and 0.43 for LG.

Another potential 2R scenario that passed the tests, though at slightly lower probability, was with recoverin diverging from the other three isoforms at 1R. However, in this case support within the recoverin clade dropped to approximately 75%, which is suggestive of a problem. For the two possible combinations of positions of the agnathan clades, and for both substitution models, we obtained ΔlogL ≈ 7, and the tree passed the three tests with *p*-AU ≈ 0.2–0.3. Therefore, we cannot exclude the possibility that recoverin diverged from the other three isoforms at 1R. Two scenarios that failed our tests of topology (though only marginally) involved expansion occurring only at the second round of WGD, so that each agnathan clade is sister to a jawed vertebrate clade. Both combinations of such pairings yielded ΔlogL ≈ 10, and both failed at least two of the tests, for the WAG and LG substitution models, though *p*-AU was close to 0.05.

#### 2.3.2. Pattern of gene duplications and losses for recoverin and visinin genes

In the light of this analysis, our preferred scenario for the origin of the four clades is presented in figure 3*b*, where the position of visinin is indicated by the heavy dashed arrow, and corresponds to the position shown for LG26 in figure 2. However, although this topology is the most plausible of the possible 2R models, on the basis of its smallest ΔlogL and largest *p*-AU, we cannot rule out the possibility that jawed vertebrate visinin is instead sister to one or other of the two agnathan isoforms, as indicated by the two dotted arrows in figure 3*b*. In order to distinguish between these cases, we would need a conclusive means of assigning the gene groupings for visinin in figure 2 (spotted gar LG26 and zebrafish ZF3 and ZF19) to either the green or red rows in figure 1.

Inspection of the fully expanded tree in electronic supplementary material, figure S2 shows the absence of any avian or reptilian taxa in the sub-tree for recoverin, leading us to conclude that recoverin has been lost from sauropsids; it is presumably because of this loss that most of the sauropsid visinins in the NCBI database are incorrectly annotated as recoverins. For cartilaginous fish species, we were unable to identify any visinins, either in our transcriptomes (table 1; electronic supplementary material, table S3) or in the NCBI database. Inspection of the sub-tree for visinin in electronic supplementary material, figure S2 shows the absence of mammalian and cartilaginous fish species, and we conclude that the visinin gene been lost from those two lineages.

As indicated by the red and blue colour in figure 3, we propose that the ancestral jawed vertebrate used visinin in its cones and recoverin in its rods. This arrangement is retained in frog [22], but the situation has not, to our knowledge, been investigated in other ‘non-3R’ taxa. We presume that those taxa that have lost one of the genes use the other isoform in both classes of photoreceptor.

#### 2.3.3. Structure of recoverin/visinin in lampreys and cartilaginous fish

For the eight lamprey proteins in the recoverin/visinin family (four encoded by our transcripts, and four from *P. marinus* and *L. camtschaticum*), we examined the key sites for Ca^2+^ binding in the four EF hands. Table 2 shows that, for all of the agnathan sequences, EF2 and EF3 possess canonical Ca^2+^-binding sites formed by acidic residues for Ca^2+^ coordination, whereas EF1 and EF4 lack the key residues for Ca^2+^ binding. In fact, the residues at the critical positions 1, 3, 5 and 12 [66] were completely conserved, as Asp, Asn, Asp, Glu in EF2, and as Asp, Asp, Asn, Glu in EF3, across every member of the recoverin/visinin family and also the outgroup (see electronic supplementary material, file S1), with the sole exception of a single Asn/Asp substitution in one of the pair of zebrafish recoverins. Thus, for every recoverin/visinin sequence that we examined, from lampreys as well as jawed vertebrates, four EF hands were present, but only EF2 and EF3 could bind Ca^2+^.

**Table 2.** Residues in EF-hands, for recoverin/visinin and GCAP/GCIP sequences. Residues are shown for positions 1–12 in the loop region of EF-hands 1–4 for selected sequences that include all those we have for agnathan species. Members of the recoverin/visinin clades are shown above, and members of the GCAP/GCIP clades are shown below. Residues are shown in bold and coloured at positions 1, 3, 5 and 12 for those cases where the EF-hand is predicted to bind Ca^2+^. The requirements for Ca^2+^ binding are that the residues at these positions should be: D, (D or N), (D or N or S) and E, respectively.

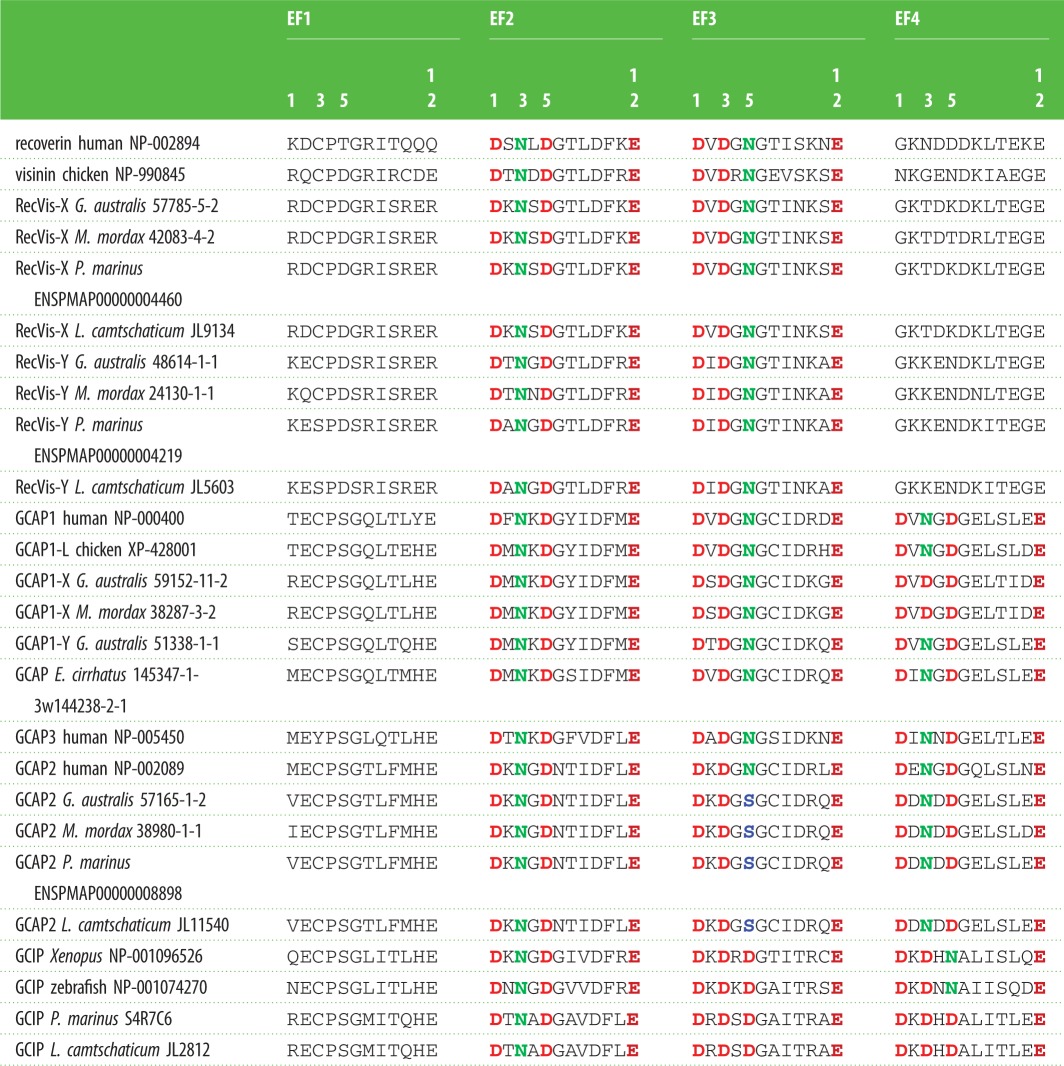

In addition, we modelled the predicted protein structure against a common recoverin template and, as shown in electronic supplementary material, figure S3, each protein generated the expected structure. As in mammalian recoverin, there were two domains (N-terminal and C-terminal), with each domain containing two EF-hand motifs forming a helix-loop-helix structure with two perpendicularly placed α-helices and a connecting loop. We therefore conclude that all of these recoverin/visinin proteins are likely to be fully functional, and to perform roles in phototransduction similar to the roles reported for recoverin and visinin in other species.

The upper section of table 1 summarizes the transcript levels that we detected for the pairs of recoverin/visinin isoforms in our two species of lamprey. In *M. mordax*, which has only a single class of photoreceptor and which expresses only the LWS opsin, we found transcripts for both isoforms, though with the level for RecVis-X around sixfold higher than for RecVis-Y. In *G. australis*, which has five classes of retinal photoreceptor and five opsins, the ratio was even greater, at more than 40-fold. Thus, it would seem likely that each class of lamprey photoreceptor expresses both isoforms of recoverin/visinin, but with a low level of RecVis-Y, although it may reflect restriction of RecVis-Y to one of the less common classes of photoreceptor.

### 2.4. Guanylyl cyclase activating proteins, GCAPs

#### 2.4.1. Syntenic arrangement of the genes encoding GCAPs

The syntenic arrangement of spotted gar genes neighbouring those encoding the GCAPs was presented in figure 1*e*, and the corresponding arrangements in human and chicken are presented in electronic supplementary material, table S1. As mentioned previously, the genes encoding GCAP1 and GCAP2 are arranged tail-to-tail in this and many other species. A striking feature of figure 1*e* is the close proximity of this family of genes to the genes for the visual opsins and GNAI/GNAT in figure 1*f*. Thus, on spotted gar LG3, GCAP1 and GCAP2 are within 4 Mb of Rh2 and GNAT2, and correspondingly, on LG8, GCAP1-L is within 3 Mb of SWS1. Because of this proximity, we hypothesize that all of these gene families formed part of a single ancestral paralogon.

The location of the GCAP3 gene (*GUCA1C*) on the same chromosome (LG3) as the GCAP1 and GCAP2 genes is unusual in spotted gar; in most genomes, GCAP3 is located on a chromosome other than the one containing GCAP1 and GCAP2 (e.g. electronic supplementary material, table S2). We were not able to identify a consistent chromosomal relationship between GCAP3 and other gene families, when examined across taxa. Thus, although we found a set of at least six other genes in the vicinity of *GUCA1C* (namely *TRAT1*, *CD47*, *NECTIN3*, *C3orf52*, *TAGLN3* and *TMPRSS7*) that were syntenic across at least six taxa (spotted gar, anole, chicken, opossum, mouse and human) as shown in electronic supplementary material, table S2, we were not able to detect a consistent pattern in their position relative to other genes. In the case of spotted gar, we hypothesize that GCAP3 (*GUCA1C*) and several nearby genes have been translocated on to LG3 from an ancestral location on another linkage group, corresponding to one of the top two rows of the quartet in figure 1. We have illustrated this hypothesis using the dotted arrow in figure 1*e*, where we suggest that the ancestral position of GCAP3 may have been on the second row; however, on the evidence available to us, it might equally have been on the top row.

We recently provided phylogenetic evidence supporting the likelihood that GNAI1 and GNAI3 diverged from GNAI2 at the first round of 2R WGD [9]. Therefore, as GCAP1 and GCAP1-L are located on the same pair of linkage groups as GNAI1 and GNAI2, we conclude from figure 1*e* that GCAP1 and GCAP1-L are very likely to have diverged from GCAP3 at 1R.

In figure 1, we have divided the seven GCAP/GCIP genes into three families (indicated by three columns), for reasons that will become clear from the phylogenetic analysis in the next section. In particular, we have placed GCIP in a family separate from GCAP2. Of the two additional GCAP2-like isoforms, we found GCAP2-A in eight taxa, but GCAP2-B only in bony fish (bowfin, spotted gar, Florida gar, zebrafish and medaka). In spotted gar, GCAP2-A and GCAP2-B are arranged head-to-tail on LG27, suggesting that the pair probably arose via a local duplication. For the only other taxon, we found (medaka) that possesses both isoforms, and that has a genome assembly, GCAP2-A and GCAP2-B are about 5 Mb apart on chromosome 3. With only two occurrences of both isoforms, there is little that we can surmise about their relationship.

#### 2.4.2. Molecular phylogeny of GCAPs

**Figure 4.**
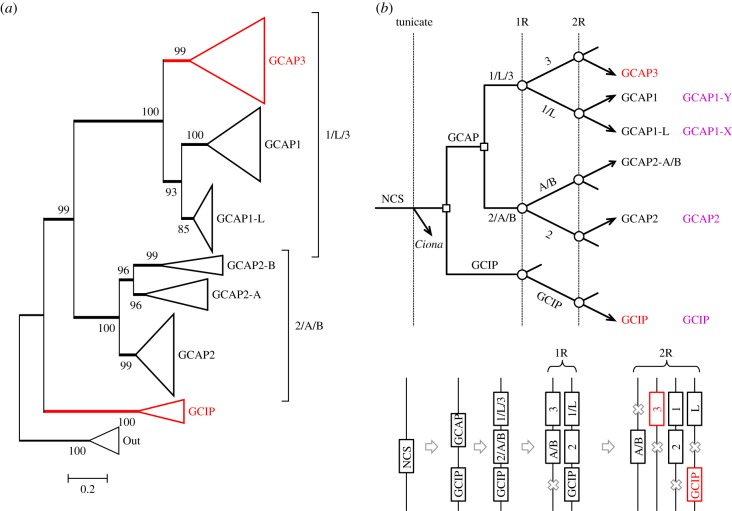
Molecular phylogeny and proposed gene duplications and losses for jawed vertebrate GCAPs. (*a*) Unconstrained molecular phylogeny for GCAP sequences from jawed vertebrates, in collapsed form; the fully expanded tree is shown in electronic supplementary material, figure S4. Support for the two main sub-trees, 1/3/L and 2/A/B, is unanimous, and support for each individual clade except one is at least 96%; GCAP1-L is supported at 85%. GCAP3 and GCIP are coloured red, as they are thought to be expressed only in cones. (*b*) Proposed scenario for gene duplications and losses. We invoke two duplications prior to WGD: a neuronal calcium sensor (NCS) duplicated to form GCIP and the ancestral GCAP, and then that GCAP duplicated to form what would become the 1/3/L and 2/A/B families. Subsequent duplications and losses occurred at 1R and 2R as indicated. The bottom panel shows the corresponding scenario for the positions of genes on blocks at the indicated times.

The multiple sequence alignment for our curated set of GCAPs is given in electronic supplementary material, file S2. Figure 4*a* presents the unconstrained molecular phylogeny for jawed vertebrate GCAPs in collapsed form, obtained using the same set of outgroup sequences that we used for recoverin and visinin in figure 3*a*; the fully expanded tree is shown in electronic supplementary material, figure S4. Six of the seven jawed vertebrate clades exhibit bootstrap support of at least 96%, and the three sub-trees are supported unanimously. The main branching pattern of ((1/L/3, 2/A/B), GCIP) is supported at a bootstrap level of 99%, and provides the basis for our depiction of GCIP on a separate column in figure 1*e*. In the absence of this phylogeny, it might have appeared appropriate to place GCIP in the same column as 2/A/B in figure 1*e*.

Within the 1/L/3 sub-tree, the placement of GCAP1 as sister to GCAP1-L is supported at a level of 93%, and this topology conforms to the paralogon arrangement that we deduced above for this set of genes. Interestingly though, when we constrained GCAP3 to be sister to GCAP1-L, we could not rule out the resulting tree on the basis of phylogeny alone, because the constrained tree showed a relatively small change in log likelihood, ΔlogL = 3.5, and it passed the three tests of topology with *p*-AU = 0.18. Thus, phylogeny on its own is not sufficient to define the topology within the GCAPs 1/L/3 sub-tree, but the combination of phylogeny and synteny provides compelling evidence.

When we additionally included lamprey sequences in the phylogeny, we initially obtained trees with rather poor bootstrap support for several clades. We think that this may have resulted from the loss of the GCAP1-L clade from all mammalian species, with the possible result that the remaining two main clades (GCAP1 and GCAP2) have been subjected to different pressures in mammals from the pressures on their orthologues in other taxa. In any case, we found that by omitting all mammalian sequences when we included lamprey sequences, we could obtain a highly plausible tree that conformed to the topology shown in figure 4 for jawed vertebrate sequences alone, and that, with a constraint, also conformed to 2R WGD followed by A-J speciation (electronic supplementary material, figure S5). Thus, the constraint ((GCAP1-X,GCAP1-L), (GCAP1-Y,GCAP1)) caused only a minor change in log likelihood, ΔlogL = 2.5, and the tree passed all tests of topology, with *p*-AU = 0.38. We also tried the alternate topology, ((GCAP1-Y,GCAP1-L), (GCAP1-X,GCAP1)), and again the tree passed all tests of topology, though with a slightly larger change in log likelihood, ΔlogL = 4.2, and with *p*-AU = 0.12; in addition, the constrained sub-tree was a tetrafurcation. We conclude that there are no grounds for rejecting the hypothesis that jawed vertebrate 1/L and agnathan X/Y clades arose in the second round of 2R WGD duplication followed by speciation, though we cannot assign orthologues unambiguously. On the other hand, in the 2/A/B sub-tree of electronic supplementary material, figure S5, there was high bootstrap support (94%) for the clade of four lamprey sequences being sister to jawed vertebrate GCAP2, so we conclude that they are orthologues and therefore we have annotated these sequences as GCAP2 (rather than as GCAP2-X).

#### 2.4.3. Pattern of duplications of GCAP genes and their subsequent loss in different lineages

By combining the analyses of synteny (figure 1*e*) and phylogeny (figure 4*a*; electronic supplementary material, figure S5), our proposed scenario for the gene duplications and losses underlying the origin of GCAPs is presented in figure 4*b*. An ancestral neuronal calcium sensor (NCS) duplicated to form GCIP and an ancestral GCAP; subsequently that GCAP duplicated locally to form the 1/L/3 and 2/A/B divisions. Although we have illustrated these first two duplications as occurring after the divergence of tunicates, there is scant evidence to determine that timing. Following the first round (1R) of genome duplication, a single gene loss occurred (in the GCIP lineage) and then after the second round (2R) four losses occurred. In conformity with our choice in figure 1*e*, we have chosen to illustrate GCAP3 on the chromosome that does not contain GCAP2-A/B, but it is possible that GCAP3 might originally have been on that other block. The magenta names in the right-hand column denote the isoforms in lampreys. The tree in electronic supplementary material, figure S5 supports the notion that each of these lamprey isoforms is orthologous with a jawed vertebrate isoform; nevertheless, we have retained the terminology GCAP1-X and GCAP1-Y until their orthology is established with more certainty.

Subsequent to their expansion during 2R WGD, various isoforms have been lost from different lineages, though GCAP2 has been retained in all the major lineages. Notably, mammals have lost GCAP1-L, which is retained in each of the other major lineages, where it forms the most highly conserved of all the GCAP clades; hence, its loss from mammals may have been very significant. Sharks and rays have lost both GCAP1 and GCAP3, and retain only GCAP1-L from the 1/L/3 group; however, the elephant shark, a chimaera, retains both. GCAP2-A and GCAP2-B are found in only a few jawed vertebrate taxa, and appear not to be present in agnathan taxa. GCIP, which appears not to have duplicates remaining from 2R, has been lost from cartilaginous fish and from amniotes.

The second section in table 1 summarizes the levels of GCAP transcripts for selected taxa. Interestingly, in both bowfin and Florida gar we found transcripts for all six jawed vertebrate isoforms, though GCAP3 and GCAP2-B were present at lower levels than the other four isoforms (GCAP1, GCAP1-L, GCAP2 and GCAP2-A). In cartilaginous fish, we detected only GCAP2 and GCAP1-L, with the former present at three to four times the level of the latter. In lampreys, it is likely that GCAP1-X is orthologous to jawed vertebrate GCAP1-L (see electronic supplementary material, figure S5), and this would provide consistency of expression levels of 1/L/3 isoforms across agnathan and cartilaginous fish taxa. If this is correct, then the GCAP isoforms expressed in the photoreceptors of lampreys and cartilaginous fish would comprise GCAP2 and GCAP1-L (=GCAP1-X), together with a trace level of GCAP1-Y (=GCAP1) in *G. australis*.

#### 2.4.4. Functional groups and structure of GCAPs in agnathan and cartilaginous fish

For each of our GCAP and GCIP sequences from agnathan, cartilaginous and basal fish, we examined the residues in the EF-hands and we modelled each protein's molecular structure. In every case, the structure could be modelled onto existing GCAP templates (electronic supplementary material, figure S6) and the critical residues in EF2–EF4 conformed to the requirements for binding Ca^2+^, as shown for the agnathan sequences in table 2. The critical locations are at sites 1, 3, 5 and 12 in each EF-hand loop, and the required residues at these sites are Asp, (Asp/Asn), (Asp/Asn/Ser) and Glu, respectively (reviewed in [66]). However, although we can be confident that each of these sequences binds Ca^2+^ at each of the three EF-hands, the subtle differences seen in the agnathan GCAP2 sequences and the GCIP sequences might impact on the properties of Ca^2+^ binding.

As mentioned previously, jawed vertebrate GCAP1 and GCAP2 both possess an N-terminal myristoyl group [35,36]. To assess the likelihood of myristoylation in agnathan GCAPs, we used the Myristoylator program (https://web.expasy.org/myristoylator). This predicted the presence of a myristoyl group in each lamprey GCAP1-X/GCAP1-Y and the absence of a myristoyl in each lamprey GCAP2. For the GCIPs, Myristoylator likewise predicted that the sequences from jawed vertebrates (e.g. *Xenopus* and zebrafish) will be myristoylated, but, as the two sequences from lampreys (*P. marinus* and *L. camtschaticum*) both lack the requisite Gly residue immediately following the N-terminal Met, it follows that these agnathan GCIPs cannot be myristoylated.

### 2.5. Guanylyl cyclases, GCs

#### 2.5.1. Syntenic arrangement of guanylyl cyclase genes

Figure 1*b* shows the arrangement of spotted gar genes in the vicinity of those encoding the guanylyl cyclases, and the corresponding arrangements in human and chicken are presented in electronic supplementary material, table S1. The most striking feature is that the three GC genes on spotted gar LG2, LG3 and LG7 appear to be part of the same paralogon identified for the arrestin and visual GRK genes [2,3]. From our recent phylogenetic analysis [10], we concluded that the two visual arrestins diverged from the two β-arrestins at 1R, and likewise that GRK7 diverged from the two GRK1s at 1R. Accordingly, the simplest interpretation of the arrangement in figure 1*b* would be that GC-F diverged from GC-D and GC-E at 1R but, as detailed below, this does not appear to be the correct interpretation.

#### 2.5.2. Molecular phylogeny of guanylyl cyclases

**Figure 5.**
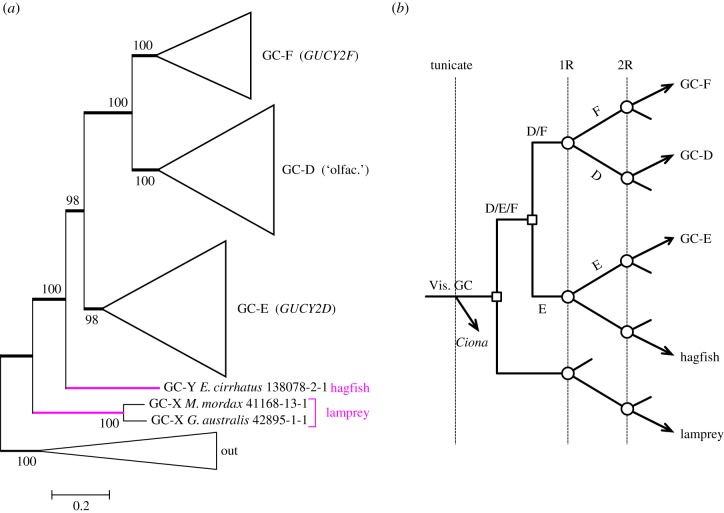
Molecular phylogeny and proposed gene duplications and losses for vertebrate visual guanylyl cyclases. (*a*) Unconstrained molecular phylogeny for GC sequences from jawed and agnathan vertebrates, in collapsed form. For an unconstrained tree, the level of support at every node is remarkably high. The fully expanded tree is shown in electronic supplementary material, figure S7, and in addition a tree with the single hagfish sequence constrained to clade with the GC-Es is given in electronic supplementary material, figure S8. GC-E (=Ret-GC1) is encoded by *GUCY2D* in human; GC-F (=Ret-GC2) is encoded by *GUCY2F*; GC-D is often referred to as ‘olfactory’, yet it is expressed in the retina in a number of aquatic taxa. (*b*) Proposed scenario for gene duplications and losses. We invoke two duplications prior to WGD. Following the duplication of an ancestral visual GC, one of the genes has been retained only in lampreys. The other duplicated to form GC-E plus the forerunner of GC-D and GC-F. The subsequent duplications during 2R WGD were followed by multiple losses.

The multiple sequence alignment for our curated set of GCs is presented in electronic supplementary material, file S3. Figure 5*a* presents the unconstrained molecular phylogeny for these visual and ‘olfactory’ GCs, from jawed and agnathan vertebrate taxa, in collapsed form; the fully expanded tree is given in electronic supplementary material, figure S7. Bootstrap support in this unconstrained tree is remarkably high, being at least 98% at every node. There is unanimous support for GC-F being sister to GC-D (and unanimous support within those two sub-trees), as well as 98% support for GC-E being sister to this pair. Therefore, given that we interpret figure 1*b* to show GC-F having diverged from GC-D at 1R, we are left with the conclusion that the GC-E and GC-D/GC-F divisions must have existed prior to the two rounds of whole-genome duplication.

To test whether the hagfish and lamprey sequences could reasonably constitute a single clade, we applied constrained tree inference and found that this possibility was decisively rejected in all three tests of topology, with *p*-AU < 0.004. On the other hand, we could not reject the possibility that the hagfish sequence was in fact a member of the GC-E clade, because the tree constrained in that way (electronic supplementary material, figure S8) passed all three tests of topology at a borderline level, with *p*-AU = 0.055.

#### 2.5.3. Pattern of duplications and losses for guanylyl cyclase genes

Our interpretation of the gene duplications and losses required to reconcile our results for synteny (figure 1*b*) and phylogeny (figure 5*a*) is presented in figure 5*b*. We propose that an ancestral ‘visual’ GC underwent local duplication twice prior to 2R WGD. Of the resulting three ‘pre-2R’ genes, the only one to have expanded during WGD was ‘D/F’, to generate GC-D and GC-F. In this proposed scheme, we need to assume extensive loss of genes, as indicated by the short lines in figure 5*b*.

Interestingly, GC-D is present in all the major lineages and in the great majority of taxa, with the notable exception of primates, where it is a pseudogene. In mouse (and presumably in other non-primate mammals), it is not expressed in the retina, but instead only in a subset of olfactory receptor neurons, where it functions as the receptor molecule for detecting CO_2_ and the proteins guanylin and uroguanylin (reviewed in [42]). By contrast, in the retina transcriptome for each of our cartilaginous and basal bony fish taxa, we found transcripts for GC-D present at substantial levels (table 1; electronic supplementary material, table S3). Indeed, in bowfin, GC-D was the principal isoform present, with a transcript level threefold higher than for GC-E; we did not detect GC-F. The most obvious explanation would be that, in these aquatic species, GC-D is expressed in retinal photoreceptors where it plays a role comparable to that played by GC-E and GC-F in mammalian cones and rods.

#### 2.5.4. Functional domains of the guanylyl cyclase proteins in agnathan taxa

Examination of the amino acid sequences for our agnathan transcripts showed that all seven of the functional domains referred to in §1.3 were present in each of the sequences (electronic supplementary material, figure S9). We next examined the degree of amino acid divergence between these functional domains, for the three agnathan sequences in comparison with a reference sequence, chosen as the human sequence in the closest clade (GC-E). Electronic supplementary material, table S4 lists the mean number of amino acid substitutions per residue, for the seven domains. Over each of the first five domains, the degree of divergence between the agnathan and human sequences is substantial (around 0.4–0.7 substitutions/residue), whereas for the final two domains (DD and CCD) the divergence is much smaller. Indeed, for the DD, the three agnathan sequences are identical, and differ by only a single residue from the human sequence. For the catalytic domain, CCD, the two lamprey sequences show a divergence from human (and from each other) of only approximately 0.1 substitutions/residue, whereas the hagfish sequence is marginally more divergent. We interpret the high level of sequence identity within these last two regions to indicate that agnathan photoreceptor GC sequences are highly likely to form dimers and to synthesize cGMP in a manner closely similar to their jawed vertebrate paralogues. We further interpret the considerably lower levels of identity in the other domains to suggest that their regulation by GCAPs might exhibit qualitative and quantitative differences from the situation in jawed vertebrates.

### 2.6. Na^+^/Ca^2+^–K^+^ exchangers, NCKXs

#### 2.6.1. Syntenic arrangement of photoreceptor NCKX genes, *SLC24A1* and *SLC24A2*

Figure 1*d* shows the arrangement of genes in the vicinity of those encoding the photoreceptor NCKXs, *SLC24A1* and *SLC24A2* in spotted gar, and the patterns in human and chicken are presented in electronic supplementary material, table S1. This arrangement strongly suggests that the duplication of the two visual NCKX genes occurred during 2R. On the other hand, we have scant evidence that the rows shown in the leftmost part of section (*d*) are continuous with the other rows shown in figure 1, and accordingly we have coloured only the third row (green) in this vicinity. Nevertheless, if the indicated order of rows is indeed preserved across the whole of figure 1, then we would anticipate that NCKX1 (*SLC24A1*) and NCKX2 (*SLC24A2*) diverged at the second round of WGD.

#### 2.6.2. Molecular phylogeny of NCKXs

In addition to jawed vertebrate NCKX1 and NCKX2 sequences, we located eight lamprey sequences (including our four transcripts) and two hagfish transcripts. Only one of the eight lamprey sequences was full length (673 residues), and although two others had at least 80% coverage (571 and 554 residues), the remaining four sequences were short fragments (of 165 to 211 residues). We included our hagfish transcript that appeared to be full-length, but we omitted a second approximately half-length hagfish transcript (see electronic supplementary material, table S3), because it was quite divergent and we could not obtain a tree with good bootstrap support when it was included. For the outgroup, we used two lancelet sequences together with human NCKX3, NCKX4 and NCKX5 (electronic supplementary material, figure S10). The multiple sequence alignment for our curated set of NCKXs is presented in electronic supplementary material, File S4.

**Figure 6.**
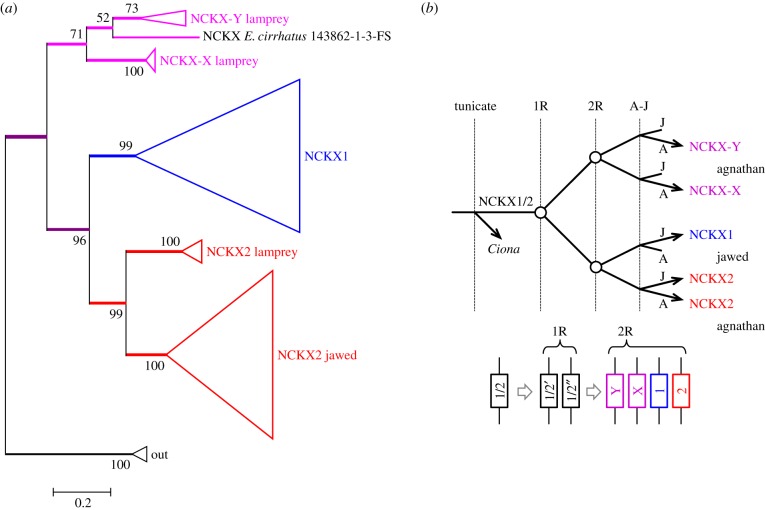
Molecular phylogeny and proposed gene duplications and losses for vertebrate visual NCKX genes. (*a*) Unconstrained molecular phylogeny for visual NCKX sequences from jawed vertebrates and lampreys, in collapsed form; the fully expanded tree is presented in electronic supplementary material, figure S11. Alignment SATé with ClustalW (see Methods). Lampreys possess three visual NCKX genes, one of which is the orthologue of jawed vertebrate NCKX2. (*b*) Proposed scenario for gene duplications and losses. The ancestral NCKX1/2 gene duplicated at 1R, and then at 2R one of these duplicated to form NCKX1 and NCKX2, whereas the other duplicated to form NCKX-X and NCKX-Y which have been lost in jawed vertebrates.

When we excluded the outgroup, the molecular phylogeny that we obtained for vertebrate visual NCKX sequences displayed unanimous support for all clades apart from the single hagfish sequences (electronic supplementary material, figure S10). With an outgroup comprising two lancelet sequences, figure 6*a* shows that the same topology was obtained for the vertebrate clades, but the levels of support dropped somewhat; the fully expanded tree is shown in electronic supplementary material, figure S11. In the view of the fact that four of the lamprey sequences were short fragments, we regard the level of support as acceptable. The lower lamprey clade (coloured red) comprised the three longest sequences (greater than 80% coverage) and was placed as sister to jawed vertebrate NCKX2 with 99% support. The other two lamprey clades (labelled NCKX-X and NCKX-Y) contained the short fragments and were positioned as sisters, and jointly as sister to the NCKX1 and NCKX2 clades.

#### 2.6.3. Pattern of duplications and losses for NCKX genes

We interpret the phylogeny in figure 6*a* to indicate the likelihood that NCKX1 and NCKX2 diverged at the second round of WGD, according to the pattern of gene duplications and losses shown in figure 6*b*. Thus, an ancestral NCKX1/2 duplicated twice during 2R WGD, without loss of genes after the first round, and with three losses after the second round. Accordingly, this interpretation (based on molecular phylogeny) supports our earlier tentative interpretation that *SLC24A1* and *SLC24A2* are located on a pair of chromosomal rows that we postulate separated from the other pair at 1R. In other words, these phylogenetic data are *consistent* with our proposal that the order of rows is maintained across figure 1, though they cannot be viewed as providing strong support for that hypothesis.

For the short-headed lamprey, *M. mordax*, which possesses LWS as the only visual opsin, table 1 shows that the only NCKX detected was the ‘cone’ isoform NCKX2. For *G. australis*, comparison of the transcript levels for eyes from juvenile downstream-migrant and adult upstream-migrant animals showed that NCKX2 was found almost exclusively in the downstream migrants (data not shown). Furthermore the other two isoforms, NCKX-X and NCKX-Y which are both expressed only at trace levels, were also found to be downstream-dominant, though to a much lesser extent than NCKX2. The downstream migrants are known to be ‘cone-dominant’, expressing higher levels of LWS, SWS1 and SWS2 opsins and lower levels of Rh1 [8,67,68]. Hence the above findings are consistent with the idea that, in *G. australis*, the cone-like photoreceptors express only NCKX2, and at moderate levels, and they also suggest that the rod-like photoreceptors may express much lower levels of the other two isoforms.

#### 2.6.4. Functional motifs and structure of the NCKX proteins of lampreys

It is difficult to form an overall picture of sequence conservation across the set of lamprey NCKX proteins because six of the eight sequences were quite short fragments, of less than half the length of jawed vertebrate NCKX2, and only a single lamprey sequence (*G. australis* NCKX2) appeared to be full-length. Key functional motifs in the NCKX proteins are two *α*-repeats that are involved in the binding and transport of Na^+^, Ca^2+^ and K^+^ in NCKX2 [69], and by homology also in NCKX1. These motifs cover the 60 residues 161–220 and 520–579 in human NCKX2, and 481–540 and 958–1017 in human NCKX1 [70]. The region of the first of these repeats is included in the partial sequences of *L. camtschaticum* (JL4915, JL8985 and JL371), and in the NCKX2 sequences of *G. australis* (full length) and *M. mordax* and *P. marinus* (partial). Across those sequences, there is only a single site that differs (Asp492Glu, for human NCKX1 versus *L. camtschaticum* JL371). In the second repeat, only the NCKX2 sequences of *G. australis* (full length) and *M. mordax* and *P. marinus* (partial) have coverage. Among these, there is again only a single site (431) that differs, with the three lamprey sequences possessing Thr and the two human sequences Ser. This analysis shows that the cation-binding/transporting region is very highly conserved.

## 3. Discussion

### 3.1. Paralogon arrangement of phototransduction cascade genes

**Figure 7.**
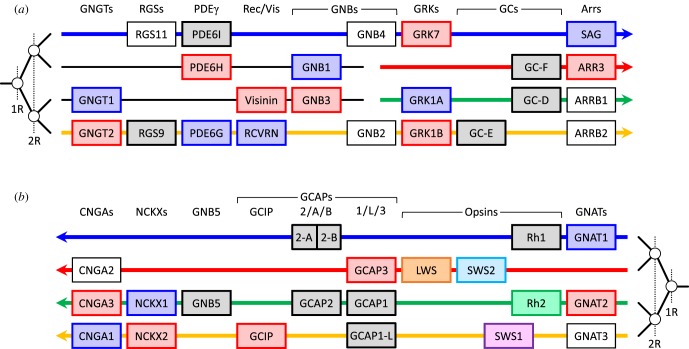
Summary of proposed ancestral arrangement of vertebrate phototransduction gene families. The genes are taken from figures 1 and 2 after omission of families not involved in phototransduction. We deduced the 1R/2R branching pattern from the combined phylogenies for the families, placing particular weight on the GRKs, arrestins and GNAIs/GNATs. For the first three families in (*a*), we cannot assign the positions of the two middle rows, and they may correspond to either the red or the green row. The genes shown in white boxes indicate paralogues that are not used in retinal photoreceptors, though *GNAT3* is used in parietal photoreceptors.

An unanticipated finding of our analysis has been the conclusion that as many as 36 genes encoding proteins directly involved in the vertebrate phototransduction cascade are contained within what appears to be a single paralogon, as summarized in figure 7. The first five gene families in figure 7*a* (GNGTs, RGS9/11, PDE6*γ*s, recoverin/visinin and GNB1–4s), comprising 10 genes involved in phototransduction, have been extracted from figure 2, where we were unable to conclusively identify which of the two middle rows should be positioned second and third. Eight of the remaining nine families, comprising 25 genes involved in phototransduction have been extracted from figure 1; in addition, as explained below, we have also included *GNB5*. In our view, it is very likely that these 14 families, comprising 36 phototransduction genes, are part of a single paralogon. This expands upon the previous proposal [2,3] that the transducin β-subunit genes *GNB1*–*GNB4* are all members of a paralogon that includes the visual opsins. In figure 7, we have split several gene families into two or more columns, on the basis of evidence for duplications prior to 2R WGD. Thus, as noted in [2], the Ensembl gene tree shows genes from protostome and tunicate taxa intervening within *GNB1*–*GNB4* and, likewise, there is evidence that the opsin duplications (apart from *Rh1*/*Rh2*) preceded 2R WGD [9]. Finally, we have presented evidence in the present paper for pre-2R duplications within the GC and GCAP/GCIP families.

Although the paralogon summarized in figure 7 contains a large number of the genes involved in vertebrate phototransduction, there are several genes missing, and we now consider other potential members. Perhaps the most obvious set of missing genes is the trio of PDE6 catalytic subunits (*PDE6A*/*B*/*C*). This family has been shown by Lagman *et al.* to reside in a paralogous region (see fig. 2 of [7]), and so the question arises as to whether that region might at some stage have been contiguous with the main paralogon. In likely support of this view are the following observations: (*a*) the genes that they describe in the vicinity of *PDE6B* (on spotted gar LG2/LG4, on human Hsa4 and on chicken Gga4) all correspond to the bottom (orange) row of figure 1, around the boundary between LG2 and LG4 in spotted gar and (*b*) the genes that they describe in the vicinity of *PDE6A* (on spotted gar LG6 and on human Hsa5) lie at what might be the corresponding position on the top (blue) row of figure 1; on the other hand, we were not able to find a region of figure 1 corresponding to *PDE6C*. If the above relationships were to prove valid, then their positions on the top and bottom rows would indicate that *PDE6A* and *PDE6B* had diverged at the first round of 2R WGD. Apart from these genes, the only other phototransduction genes that have not been accounted for in figures 1 and 2 are the CNGC channel β-subunit genes (*CNGB1*/*3*) and *RGS9BP*.

The probability that so many separate gene families (nine in figure 1) contributing to a common function would occur in such proximity as a result of random placement is very low. Excluding a few outlier genes, the illustrated regions in figure 1 encompass no more than approximately 70 Mb along each of four rows, out of a total length for the spotted gar genome assembly of approximately 900 Mb, so that each row corresponds to approximately 31% of the total available length. The probability that these nine gene families would happen to be co-located within such a region by chance can be estimated as 0.31^8^ ≈ 10^−4^, and we therefore reject the notion that such proximity occurred randomly. Instead, we conclude that prior to 2R WGD, there must have been some advantage in these ancestral genes being close together in the ‘pre-quadruplication’ genome; one such advantage might have involved the ability to regulate gene expression within the phototransduction cascade.

Our definition of the 1R/2R branching pattern in figures 1, 2 and 7 is based on our previous phylogenetic analyses of the GRKs, arrestins and GNAIs [9,10]. Those phylogenies provided high levels of support for the following isoforms being sisters: *GRK1A*/*GRK1B*, *SAG*/*ARR3* and *GNAI3*/*GNAI1* (with the last pair also implying *GNAT2*/*GNAT3*). On that basis, we conclude that the upper pair of rows (blue and red) diverged from the lower pair of rows (green and orange), with that first divergence having occurred at 1R.

That interpretation has a corollary for the identification of the gene duplications within the *CNGA* family. Previously [9], we proposed that *CNGA1* diverged from (*CNGA2*, *CNGA3*) at 1R, because the latter two clades were positioned as sisters. However, that pattern does not conform with the row positions of the *CNGA* genes shown in figures 1 and 7*a*. Instead, we suggest that *CNGA1* may have diverged prior to WGD, by a local duplication, and that *CNGA2* and *CNGA3* then diverged at 1R. It has previously been established that *CNGA4* diverged prior to WGD (and probably prior to the protostome/deuterostome split), and it now seems plausible that *CNGA1* also diverged before WGD. In the absence of compelling evidence, though, we have shown the *CNGA* genes in a single column, rather than placing *CNGA1* in a separate column.

### 3.2. Summary of gene duplications that gave rise to Ca-feedback regulation of phototransduction in extant vertebrates

**Figure 8.**
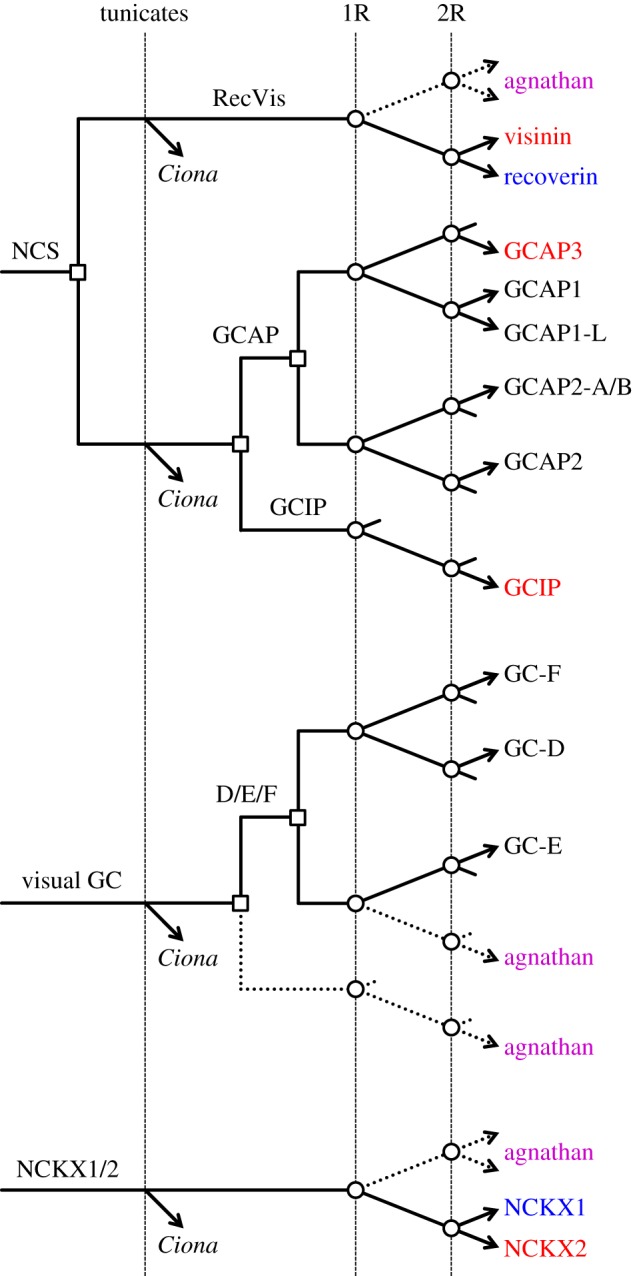
Summary of proposed patterns of duplications and losses in the families of genes encoding the proteins mediating Ca-feedback regulation of phototransduction in jawed vertebrates. NCS, neuronal calcium sensor.

In figure 8, we summarize the pattern of gene duplications (and losses) that we deduce to have given rise to the multiple isoforms in the four families of proteins mediating the Ca-feedback regulation of phototransduction in jawed vertebrates. In addition to the extensive expansion that occurred during 2R WGD, it is clear that prior to 2R WGD there were two successive gene duplications in an ancestral GCAP gene, and likewise two duplications in an ancestral visual GC gene. Within the GCAP family, the latter of these ‘pre-2R’ duplications generated the GCAP1/L/3 and GCAP2/A/B branches, and in the visual GC family the latter of the ‘pre-2R’ duplications generated the GC-E and GC-D/GC-F branches.

These last two ‘pre-2R’ gene duplications appear to have been instrumental in creating the potential for two different regimes of Ca^2+^-feedback regulation, one using a ‘GCAP1’ with GC-E and the other using a ‘GCAP2’ with a GC-D/GC-F. In the photoreceptors of extant vertebrates, it has been clearly established that such a Ca^2+^-feedback loop is crucial both to achieving a well-timed recovery of the light response and to mediating light adaptation (the ability to adjust rapidly to a change in operating intensity) [71]. Disruption of this feedback loop leads to a pronounced slowing of the recovery phase of responses to brief flashes, and also to saturation of the response at a much lower intensity of steady light than when the loop is intact. Rods recover more slowly than cones and they saturate at a much lower intensity than cones do, and while it seems likely that the different properties of the GCAP and GC isoforms expressed in rods and cones contribute to the disparities, these differences have yet to be explained in quantitative terms.

### 3.3. The origin of specialization for operation at high and low intensities

In a recent analysis of the evolution of the shut-off steps in phototransduction, we showed that the distinct isoforms of GRK expressed in cones and rods arose in a gene duplication event that occurred prior to 2R WGD, and indeed prior to the divergence of tunicates [10]. In the present paper, we show that isoforms of GCAP and isoforms of GC, which are differentially expressed in cones and rods, likewise arose through gene duplications that occurred prior to 2R WGD. Combining these observations, we conclude that multiple isoforms of three of the proteins involved in regulating response recovery and light adaptation already existed prior to the emergence of rhodopsin as a distinct visual opsin during 2R WGD. In addition, as discussed in §3.1, it is possible that separate isoforms of CNGAs also existed prior to 2R WGD. Hence we conclude that the *potential* for the specialization of proto-vertebrate photoreceptors for operation preferentially in either dim light or bright light existed before rhodopsin evolved. We further hypothesize the existence, prior to 2R WGD, of differential expression of isoforms that enabled such specialization.

Thus, we envisage one class of photoreceptor (very much like extant cones) that expressed GRK7, together with a predominance of GCAP1/L/3 and GC-E, and also possibly with CNGA2/3, that would have functioned well at high light levels. And we envisage another class of photoreceptor expressing GRK1, together with a predominance of GCAP2/A/B and GC-D/F, and also possibly with CNGA1, that would have saturated at moderate intensities, and therefore would have functioned preferentially at lower light levels.

Consistent with this idea, there has been a very recent report [72] that pinopsin evolved as the ancestral dim-light visual opsin, with a lower rate of thermal activation than cone opsins. Accordingly, it would seem likely that the ancestral low-light photoreceptor that we proposed above would have expressed pinopsin as its visual pigment. Subsequently, when rhodopsin evolved, with its very low rate of thermal isomerization and intrinsically slower shut-off, it would have been advantageous to the organism for this new visual opsin to be expressed in the ‘scotopic’ photoreceptors that we postulate already existed, thereby facilitating operation at even lower intensities than had previously been possible.

We suggest that the original impetus for a duplex arrangement of this kind may have involved the speed of the photoresponse, and in particular the speed of detection of a reduction in light intensity. When the molecular steps that mediate response recovery first evolved, it would seem likely that they would have been sluggish, either because the enzymatic efficacy of the reactions had not yet been optimized, or because the levels of protein expression were low. And, because the efficacy of these shut-off steps sets the speed for detection of a drop in light intensity, it seems likely that there would have been selective pressure to accelerate those steps, and thereby provide more rapid detection of (for example) the shadow of a predator. However, an inevitable trade-off with faster shut-off steps would have been a reduced ability to detect the onset of a dim light. Hence, there might well have been a major advantage in separating the two functions, by using one class of photoreceptor with faster shut-off reactions for the rapid detection of a drop in intensity at moderate light levels, and another class of photoreceptor with slower shut-off reactions for detection tasks at lower light levels. Pressures of this kind would have been present long before 2R WGD, and so it is perfectly natural to think that a duplex arrangement of retinal photoreceptors might have evolved long before the emergence of rhodopsin as a specialized scotopic visual pigment.

### 3.4. Issues with gene naming and annotation

As an example of the issues associated with gene naming and annotation for the multiple isoforms of phototransduction genes, we will examine the situation for the visual guanylyl cyclase genes in zebrafish. Inspection of the phylogeny in electronic supplementary material, figure S7 shows that for each of the three clades (GC-E, GC-D and GC-F) only a single 3R duplicate has been retained in zebrafish, and this simplifies the identification of gene orthology. The assignment that can be seen in electronic supplementary material, figure S7, of zebrafish isoforms to the three clades, is set out in table 3. Of the three zebrafish gene names in ZFIN, only *gc2* is orthologous with the corresponding name used in mammals, in this case RetGC-2 = GC-F. Zebrafish *gc3* (zatoichi) is orthologous with GC-E = RetGC-1, while zebrafish *gucy2f* is orthologous with GC-D, which is commonly referred to as the olfactory GC.

**Table 3.** Identification of isoforms of visual guanylyl cyclases in zebrafish.

isoform (IUPHAR/BPS)	mammalian name	ZDB-GENE-	ZF chromosome	ZF mRNA NM_	ZF protein NP_	ZF gene symbol
GC-E	RetGC-1	011128-9	5, 38.1 Mb	131866	571941	*gc3*
GC-D	Olfactory GC	011128-7	15, 29.4 Mb	131864	571939	*gucy2f*
GC-F	RetGC-2	011128-8	7, 51.3 Mb	001109695	001103165	*gc2*

Thus, we concur with Collery & Kennedy [73] that *gc3* corresponds to human *GUCY2D* = GC-E. But we reject the contention of Stiebel-Kalish *et al.* [74] that the zebrafish gene *gucy2f* on chromosome 15 is the orthologue of human *GUCY2D*; instead, it is the orthologue of GC-D, the ‘olfactory’ GC in mammals, which is expressed in the retina of many aquatic species, but which has been lost from human. Nor can we concur with the claim of Rätscho *et al.* [24] that all three of *gc2*, *gc3* and *gucy2f* show the highest degree of relationship to mammalian GC-F.

### 3.5. Significance and future directions

Overall, we regard our analyses as significant in the following ways. First, we have discovered that the great majority of vertebrate phototransduction genes are located within a single paralogon. Second, we have elucidated the gene duplication patterns for the multiple families of genes that mediate the crucial Ca-feedback loop of phototransduction, identifying several duplications prior to, and multiple duplications during, 2R WGD. Third, by examining the pre-2R duplications, we have found evidence for the likely existence of a duplex photopic/scotopic specialization in a proto-vertebrate organism before the two rounds of whole-genome duplication, and hence prior to the emergence of rhodopsin. Fourth, inspection of our phylogenetic clades has identified, more clearly than previously, those isoforms that have been lost in different lineages (e.g. the loss of visinin from mammals and cartilaginous fish, and the loss of recoverin from sauropsids). Fifth, such inspection has also identified a substantial number of misleading and/or erroneous annotations in the published sequence databases (e.g. in NCBI, ZFIN and Ensembl); accordingly, the annotation of purported rod/cone isoforms in existing databases should be treated with considerable caution.

For the future, we suggest that it will be important to confirm the positions of the sections of rows in figures 1, 2 and 7 that we have not been able to assign with certainty; this may be possible through examination of synteny in additional species, and by more extensive examination of the regions adjacent to each of the relevant gene families. It will also be important to investigate more comprehensively whether any of the few other genes that play a role in vertebrate phototransduction may additionally be associated with the ‘phototransduction paralogon’. In addition, we suggest that effort should be devoted to tabulating a revised set of names and descriptions for the genes encoding the multiple isoforms of phototransduction proteins in different species. And now that an assembly for the genome of the inshore hagfish (*E. burgeri*) is included in Ensembl, we suggest that it may be possible to obtain improved phylogenies for all agnathan clades, for which a considerable limitation has until now stemmed from the occurrence of ‘single taxon clades’, because in most cases we had transcripts for only a single species of hagfish, *E. cirrhatus*.

## 4. Methods

### 4.1. Analysis of gene synteny

We searched for paralogous gene regions, primarily from the genome of spotted gar (*Lepisosteus oculatis*), using a combination of Ensembl (www.ensembl.org), Genomicus (www.genomicus.biologie.ens.fr) and Synteny Database (syntenydb.uoregon.edu/synteny_db). Once we had located potentially interesting genes in the vicinity of phototransduction genes, we examined paralogues using Ensembl's gene tree with viewing option ‘View paralogues of current gene’. In order for a set of genes to be considered ‘2R paralogous’, we required that the Ensembl gene tree not show any invertebrate taxa (e.g. protostome or basal deuterostome species) within the set. For the sets of non-phototransduction genes in figure 1, we have illustrated only those for which we found at least three paralogues (with the exception of *KNCV2*/*L*). In addition, we have aimed to use only gene families that are reasonably close to a phototransduction gene family. Thus, 80% of the sets of non-phototransduction genes (43 of 54 sets) have at least one member within 1.7 Mb of a phototransduction gene in spotted gar, and only two sets exceed a separation of 10 Mb (*DAPK* at 11.2 Mb, and *C2CD4* at 10.1 Mb).

In assigning continuity of the rows shown in bold and coloured (blue, red, green and orange) in figure 1, we examined the locations of orthologues across three taxa (spotted gar, human and chicken), as tabulated in electronic supplementary material, table S1. Where breaks occurred, we looked for signs of synteny across taxa. Our rationale for choice of rows included the following considerations:

(1) Across the top row, the blue regions are located on LG14 and LG5 in spotted gar, and where orthologues occur in human the great majority (22 of 26) are located on chromosome 3.
(2) Across the second row, the red regions are located on LG7 and LG1 in spotted gar, and of the 27 orthologues found in human all but one are located on the X chromosome.
(3) Across the third row, the green regions are located on LG17 and LG3 in spotted gar, and in chicken all 27 orthologues of genes in panels (*a*–*c*) are located on chromosome 1, all nine orthologues for panel (*d*) are located on chromosome 10, and all 16 orthologues for panels (*e*,*f*) are located on chromosome 26.
(4) Across the fourth row, the orange regions are located on LG2 and LG8 in spotted gar, while in human five orthologues and eight orthologues, respectively, from these linkage groups are located close together on chromosome 7.

Finally, we were not able to find any swapping of regions, from the rows illustrated in figure 1, that provided a more plausible arrangement for the outcome of a presumed quadruplication followed by a limited degree of rearrangement.

### 4.2. Transcriptome data

The methods for obtaining the eye transcriptomes from basal vertebrate species were described in [8], and here we use transcripts from that work. Sequences were available for each of the following species obtained from Australian waters: *Eptatretus cirrhatus*, broad-gilled hagfish; *Geotria australis*, pouched lamprey; *Mordacia mordax*, short-headed lamprey; *Aptychotrema vincentiana*, western ray; *Aptychotrema rostrata*, eastern ray; *Neotrygon kuhlii* (*N. australiae*), bluespot ray; *Chiloscyllium punctatum*, bamboo shark; and *Carcharhinus amblyrhynchos*, reef shark. Sequences were also obtained from bowfin, *Amia calva*; and Florida gar, *Lepisosteus platyrhincus*. Searching of our transcriptomes was performed using a custom program, TriPyGDU [8], and augmented using a BLAST server, SequenceServer [75]. Here we report 73 new sequences, which have been submitted to GenBank and assigned nucleotide accession numbers MH577347-MH577419.

### 4.3. Sequence selection

We tried to use as uniform a set of taxa as possible, aiming to select: two placental mammals (human and cattle); two marsupials; three birds; three reptiles; two amphibians; bowfin and gar; two sharks; two rays; and elephant shark (a chimaera). For eastern and western ray, the orthologous sequences were identical (or nearly so) when we had both, and in those cases we used only the western ray sequence. Likewise, for the two species of gar, we used only the Florida gar sequence when we had nearly identical orthologues. For agnathan vertebrates, we used every available sequence, except for those partial sequences that we deemed to be too short. For several partial sequences, we noticed a deterioration of the alignment near the end of the sequence. In these cases, we removed the poorly aligned terminal residues; these sequences are listed as ‘-Trimmed’ in the figures in the electronic supplementary material. For outgroups, we searched for closely similar sequences from tunicates (*Ciona intenstinalis* and *C. savigni*), lancelets (*Branchiostoma floridae* and *B. belcheri*), and from two other more basal deuterostomes (*Strongylocentrotus purpuratus*, an echinoderm, and *Saccoglossus kowalevskii*, a hemichordate).

### 4.4. Multiple sequence alignment

We performed multiple sequence alignment of protein sequences using SATé-II v. 2.2.7 [76]. For the illustrated phylogenies we standardized on the following settings: aligner, MAFFT; merger, MUSCLE; tree estimator, FASTTREE; model, WAG + G20; decomposition, centroid; maximum sub-problem size, 12. To avoid introducing bias, we did not manually adjust any alignments, and we always used the entire alignment. For the NCKX sequences, we encountered a problem in that the alignment appeared to vary greatly in response to small changes (such as omission of a single sequence, or even minor trimming of a sequence); in this case, we found that using ClustalW as the aligner in SATé-II gave what appeared to be a better alignment and a tree that exhibited high support. The alignments we obtained are presented in electronic supplementary material, files S1–S4.

### 4.5. Tree inference

We constructed unconstrained maximum-likelihood (ML) phylogenetic trees using IQ-Tree (Windows multicore v. 1.5.6) [77], using the ultrafast bootstrap approximation [78]. For the phylogenies presented, we standardized on the following settings: 10 000 bootstrap replicates; protein substitution model, WAG [79]. We generally obtained very similar results using the LG substitution model [80], but these are not illustrated. Numbers at each node represent percentage bootstrap support.

Constrained trees were constructed using the ‘-g’ constraint option in IQ-Tree. In specifying the constraints, we used the minimum set of sequences that would constrain the tree as we intended. Typically, we used just a single sequence representative of the relevant isoform, and we relied on the tightness of clading to constrain the other orthologues in the same manner. Each constraint tree that we used is shown as an inset by the constrained tree. One point to bear in mind when examining constrained trees is that the level of bootstrap support at any node that has been constrained is necessarily (i.e. artificially) increased, in many cases to 100%, because of the constraint.

For each constrained tree obtained, we conducted tree topology tests using the ‘-z’ option in IQ-Tree, in order to test whether or not the constrained tree needed to be rejected in comparison with the unconstrained ML tree. The tests applied were *bp*-RELL, *c*-ELW and *p*-AU, representing, respectively, the bootstrap proportion test using the RELL method [81], the expected likelihood weight test [82] and the approximately unbiased test [83]. Only those trees that passed all tests at the 95% confidence level (i.e. *p* ≥ 0.05) were considered further.

### 4.6. Molecular modelling

The structure of proteins was predicted using SWISS-MODEL (swissmodel.expasy.org [84]). Protein sequences were used to search for appropriate templates; the same template was then used for each sequence in the class. The N-terminal myristoylation of proteins was predicted by the Myristoylator program (web.expasy.org/myristoylator). The program uses ensembles of neural networks to learn to discriminate positive and negative sequences for myristoylation.

## Supplementary Material

Recoverin/Visinin MSAs

## Supplementary Material

GCAP MSAs

## Supplementary Material

GC MSAs

## Supplementary Material

NCKX MSAs

## Supplementary Material

Supplementary Tables

## Supplementary Material

Supplementary Figures

## Data accessibility

The data supporting the findings of this article have been uploaded as part of the supplementary material. mRNA sequences have been deposited in GenBank with accession numbers MH577347-MH577419. The sets of reads from which the transcriptomes were assembled have been deposited in the NCBI Sequence Read Archive under SRA Study SRP062082.
