# Supplementary material for: Evolution of the calcium feedback steps of vertebrate phototransduction
Source: Open Biol. 2018 Sep 26;8(9):180119. doi: 10.1098/rsob.180119 (PMC6170504; doi:10.1098/rsob.180119)
Supplement: Supplementary Tables [file rsob180119supp5.pdf]

# Supplementary Tables

for *Open Biology*, http://dx.doi.org/10.1098/rsob.180119

## Evolution of the calcium feedback steps of vertebrate phototransduction

Trevor D. Lamb[1] and David M. Hunt[2,3]

[1]Eccles Institute of Neuroscience, John Curtin School of Medical Research, The Australian National University, ACT 2600, Australia, and [2]Centre for Ophthalmology and Visual Science, The Lions Eye Institute and [3]School of Biological Sciences, The University of Western Australia, WA 6009, Australia.

### Contents

| | Page |
|---|---|
| **Supplementary Table S1**. Synteny of phototransduction genes in spotted gar, human and chicken. | 2 |
| **Supplementary Table S2**. Gene synteny in the vicinity of GCAP3 (*GUCA1C*). | 5 |
| **Supplementary Table S3**. Summary of transcripts examined. | 6 |
| **Supplementary Table S4**. Sequence divergence for functional domains of GCs. | 8 |

**Supplementary Table S1**. Synteny of phototransduction genes in spotted gar, human and chicken.

Columns ‘LG’, ‘Hsa’, ‘Gga’ give chromosome numbers for spotted gar, human and chicken.
Columns ‘Mb’ give gene location in megabases.
‘–’: Gene missing from all three taxa; ‘-’: Gene missing from this taxon; ‘?’: Uncertain.

| Gene | LG | Mb | Hsa | Mb | Gga | Mb | Gene | LG | Mb | Hsa | Mb | Gga | Mb | Gene | LG | Mb | Hsa | Mb | Gga | Mb | Gene | LG | Mb | Hsa | Mb | Gga | Mb |
|---|---|---|---|---|---|---|---|---|---|---|---|---|---|---|---|---|---|---|---|---|---|---|---|---|---|---|---|
| PLOD3 | 2 | 61.6 | 7 | 101.2 | - | | – | | | | | | | PLOD1 | 25 | 9.7 | 1 | 11.9 | 21 | 5.5 | PLOD2 | 14 | 14.9 | 3 | 146.1 | 9 | 11.5 |
| CHD3 | 2 | 61.0 | 17 | 78.8 | - | | CHD4 | 26 | 12.6 | 12 | 6.6 | 1 | 77.0 | CHD5 | 25 | 8.9 | 1 | 6.1 | 21 | 6.3 | – | | | | | | |
| SLC2A4 | 2 | 60.5 | 17 | 7.3 | - | | SLC2A3 | - | | 12 | 7.9 | 1 | 75.9 | SLC2A1 | 25 | 7.4 | 1 | 42.9 | 21 | 6.5 | SLC2A2 | 14 | 13.7 | 3 | 171.0 | 9 | 19.8 |
| – | | | | | | | CLSTN3 | 26 | 12.6 | 12 | 7.1 | 1 | 77.4 | CLSTN1 | 25 | 12.5 | 1 | 9.7 | 21 | 3.5 | CLSTN2 | 14 | 7.4 | 3 | 139.9 | 9 | 6.2 |
| GNB2 | 2 | 58.9 | 7 | 100.7 | - | | **GNB3** | 26 | 12.6 | 12 | 6.8 | 1 | 77.3 | **GNB1** | 25 | 11.4 | 1 | 1.8 | 21 | 1.9 | GNB4 | 14 | 1.6 | 3 | 179.4 | 9 | 17.5 |
| STAG3 | 2 | 57.2 | 7 | 100.1 | - | | STAGL | 17 | 2.0 | - | | 1 | 130.0 | STAG2 | 7 | 39.0 | X | 124.0 | 4 | 15.6 | STAG1 | 14 | 14.3 | 3 | 136.3 | 9 | 0.7 |
| FGF11 | 2 | 60.2 | 17 | 7.4 | - | | FGF14 | 17 | 2.8 | 13 | 101.7 | 1 | 143.3 | FGF13 | 7 | 46.5 | X | 138.6 | 4 | 4.8 | FGF12 | 14 | 19.0 | 3 | 192.1 | 9 | 13.4 |
| – | | | | | | | ZIC2 | 17 | 3.4 | 13 | 100.0 | 1 | 144.3 | ZIC3 | 7 | 46.3 | X | 137.6 | 4 | 4.6 | ZIC1 | 14 | 14.7 | 3 | 147.4 | 9 | 11.9 |
| – | | | | | | | RAP2A | 17 | 4.1 | 13 | 97.4 | 1 | 145.3 | RAP2C | 7 | 45.5 | X | 132.2 | 4 | 3.5 | RAP2B | 14 | 2.9 | 3 | 153.2 | 9 | 23.4 |
| GPC2 | 2 | 57.3 | 7 | 100.2 | - | | GPC6 | 17 | 12.8 | 13 | 93.2 | 1 | 146.5 | GPC4 | 7 | 45.6 | X | 133.3 | 4 | 3.8 | GPC1 | 14 | 10.5 | 2 | 240.4 | 9 | 2.7 |
| – | | | | | | | SLC9A7 | 17 | 20.6 | X | 46.6 | 1 | 10.3 | SLC9A6 | 7 | 45.9 | X | 136.0 | 4 | 4.2 | SLC9A9 | 14 | 15.2 | 3 | 143.3 | 9 | 10.7 |
| ATP1B2 | 2 | 59.1 | 17 | 7.6 | - | | ATP4B | 17 | 27.5 | 13 | 113.6 | 1 | 137.3 | ATP1B4 | 7 | 47.3 | X | 120.4 | 4 | 16.5 | ATP1B3 | 14 | 15.5 | 3 | 141.9 | 9 | 10.3 |
| SOX19 | 2 | 63.7 | - | | - | | SOX1 | 17 | 24.5 | 13 | 112.0 | 1 | 139.8 | SOX3 | 7 | 46.8 | X | 140.5 | 4 | 10.6 | SOX2 | 14 | 0.2 | 3 | 181.7 | 9 | 16.9 |
| CD68 | 2 | 57.5 | 17 | 7.6 | - | | LAMP1 | 17 | 27.3 | 13 | 113.3 | 1 | 137.5 | LAMP2 | 7 | 47.3 | X | 120.4 | 4 | 16.5 | LAMP3 | 14 | 0.4 | 3 | 183.1 | 9 | 16.4 |
| GRK1B | 2 | 60.7 | - | | ? | | GRK1A | 17 | 27.5 | 13 | 113.7 | - | | – | | | | | | | GRK7 | 14 | 15.6 | 3 | 141.8 | 9 | 10.3 |
| SERPINE1 | 2 | 61.3 | 7 | 101.1 | - | | SERPINE3 | 17 | 29.1 | 13 | 51.3 | 1 | 170.1 | – | | | | | | | SERPINE2 | 14 | 6.6 | 2 | 224.0 | 9 | 8.3 |
| **GUCY2D** | 2 | 59.0 | 17 | 8.0 | - | | **GC–D** | 3 | 0.5 | - | | 1 | 193.6 | **GUCY2F** | 7 | 37.6 | X | 109.4 | - | | – | | | | | | |
| ARRB2 | 2 | 57.3 | 17 | 4.7 | - | | ARRB1 | 3 | 2.5 | 11 | 75.3 | ? | | **ARR3** | 7 | 46.8 | X | 70.3 | 4 | 1.3 | **SAG** | 14 | 18.8 | 2 | 233.3 | 9 | 1.2 |
| DLG4 | 2 | 59.6 | 17 | 7.2 | - | | DLG2 | 3 | 7.7 | 11 | 83.5 | 1 | 141.0 | DLG3 | 7 | 47.0 | X | 70.4 | 4 | 2.5 | DLG1 | 14 | 1.9 | 3 | 197.0 | 9 | 12.5 |
| – | | | | | | | P2RY2 | 3 | 9.7 | 11 | 73.2 | 1 | 195.4 | P2RY4 | 7 | 46.8 | X | 70.3 | 4 | 1.2 | – | | | | | | |
| SHISA3 | 4 | 57.7 | 4 | 42.4 | 4 | 68.7 | SHISA2 | 3 | 5.2 | 13 | 26.0 | 1 | 177.1 | SHISA1 | 7 | 40.1 | - | | 4 | 11.7 | – | | | | | | |
| LNX1 | 4 | 72.9 | 4 | 53.5 | 4 | 66.1 | LNX2 | 3 | 5.6 | 13 | 27.5 | 1 | 176.2 | LNX2B | 7 | 47.2 | - | | 4 | 12.2 | – | | | | | | |
| RASL11B | 4 | 72.7 | 4 | 52.9 | 4 | 66.4 | RASL11A | 3 | 5.5 | 13 | 27.3 | 1 | 176.3 | RASL11L | 7 | 37.7 | - | | 4 | 12.3 | – | | | | | | |
| USP46 | 4 | 72.6 | 4 | 52.6 | 4 | 66.4 | USP12 | 3 | 5.5 | 13 | 27.1 | 1 | 176.4 | USP12B | 7 | 37.7 | - | | 4 | 12.3 | – | | | | | | |
| KDR | 4 | 73.2 | 4 | 55.1 | 4 | 65.6 | FLT1 | 3 | 5.7 | 13 | 28.3 | 1 | 175.8 | KDRL | 7 | 47.1 | - | | 4 | 12.0 | FLT4 | 6 | 36.8 | 5 | 180.6 | 13 | 13.3 |
| KIT | 4 | 73.1 | 4 | 54.7 | 4 | 65.7 | FLT3 | 3 | 5.7 | 13 | 28.0 | 1 | 176.0 | – | | | | | | | CSF1R | 6 | 30.9 | 5 | 150.1 | 13 | 13.3 |

| | | | | | | | | | | | | | | | |
|---|---|---|---|---|---|---|---|---|---|---|---|---|---|---|---|
| SLC7A2 | 4 71.2 | 8 17.5 | 4 63.4 | SLC7A1 | 17 16.2 | 13 29.5 | 1 175.5 | SLC7A3 | 7 44.0 | X 70.9 | 4 2.4 | SLC7A2.2 | 6 34.9 | - | 13 13.0 |
| GRIA2 | 4 20.8 | 4 157.2 | 4 21.6 | GRIA4 | 17 10.3 | 11 105.6 | 1 181.2 | GRIA3 | 7 38.7 | X 123.2 | 4 15.8 | GRIA1 | 6 23.3 | 5 153.5 | 13 12.3 |
| CLCN3 | 4 63.3 | 4 169.6 | 4 25.5 | CLCN4 | 17 18.7 | X 10.2 | 1 124.8 | CLCN5 | 7 37.2 | X 49.9 | 4 9.6 | – | | | |
| GABRA2 | 4 55.4 | 4 46.2 | 4 67.5 | GABRA5 | 17 21.3 | 15 26.9 | 1 131.3 | GABRA3 | 7 22.4 | X 152.2 | 4 10.8 | GABRA1 | 6 28.9 | 5 161.8 | 13 7.2 |
| GABRB1 | 4 47.8 | 4 47.0 | - | GABRB3 | 17 21.4 | 15 26.5 | 1 131.4 | GABRB4 | 7 22.2 | X 152.6 | 4 10.7 | GABRB2 | 6 29.0 | 5 161.3 | 13 7.3 |
| TEC | 4 50.5 | 4 48.1 | 4 66.9 | BMX | 17 19.7 | X 15.5 | 1 122.0 | BTK | 7 47.9 | X 101.3 | 4 2.0 | ITK | 6 23.6 | 5 157.1 | 13 11.4 |
| **CNGA1** | 4 50.6 | 4 47.9 | 4 67.0 | **CNGA3** | 17 21.7 | 2 98.3 | 1 132.0 | CNGA2 | 7 44.6 | X 151.7 | 4 11.0 | – | | | |
| | | | | CNGA4 | 17 16.7 | 11 6.2 | 1 194.5 | | | | | | | | |
| NFIB | 4 46.7 | 9 14.1 | Z 31.4 | – | | | | NFIC | 19 2.3 | 19 3.3 | 28 1.1 | NFIA | 6 16.3 | 1 60.9 | 8 27.1 |
| HMG20L | 4 36.5 | - | - | HMG20A | 3 46.3 | 15 77.4 | 10 2.9 | HMG20B | 19 9.0 | 19 3.6 | 28 1.0 | – | | | |
| **SLC24A2** | 4 45.0 | 9 19.5 | Z 33.8 | **SLC24A1** | 3 42.9 | 15 65.6 | 10 18.1 | – | | | | – | | | |
| SH3GL2 | 4 50.1 | 9 17.6 | Z 33.0 | SH3GL3 | 3 38.6 | 15 83.4 | 10 11.4 | SH3GL1 | 19 9.6 | 19 4.4 | 28 2.5 | – | | | |
| LINGO2 | 4 52.1 | 9 27.9 | Z 68.8 | LINGO1 | 3 46.1 | 15 77.6 | 10 2.7 | LINGO3 | 19 8.6 | 19 2.3 | 28 2.0 | LINGO4 | - | 1 151.8 | - |
| ONECUT2 | 2 9.7 | 18 57.4 | Z 0.4 | ONECUT1 | 3 40.5 | 15 52.8 | 10 8.6 | ONECUT3 | 19 8.7 | 19 1.8 | 28 2.3 | ONECUTL | 24 6.8 | - | - |
| – | | | | **GNB5** | 3 40.3 | 15 52.1 | 10 8.9 | – | | | | – | | | |
| ATP8B5 | 2 11.3 | - | Z 8.9 | ATP8B4 | 3 39.3 | 15 49.9 | - | – | | | | ATP8B2 | 24 6.0 | 1 154.3 | 25 2.2 |
| C2CD4CL | 2 34.8 | - | - | C2CD4A | 3 53.0 | 15 62.1 | 10 4.4 | C2CD4C | 19 9.4 | 19 0.4 | 28 2.7 | C2CD4D | - | 1 151.8 | - |
| DAPK1 | 2 47.1 | 9 87.5 | Z 41.3 | DAPK2 | 3 54.1 | 15 63.9 | 10 3.8 | DAPK3 | 19 3.5 | 19 4.0 | 28 1.5 | DAPKL | 24 5.7 | - | 25 1.4 |
| HCN1 | 2 49.7 | 5 45.3 | Z 14.4 | HCN4 | 3 51.1 | 15 73.3 | 10 2.0 | HCN2 | 19 7.6 | 19 0.6 | 28 2.7 | HCN3 | 24 5.0 | 1 155.3 | 25 2.2 |
| KCNV2 | 2 53.4 | 9 2.7 | Z 26.6 | | | | | KCNV2L | 19 4.3 | - | 28 0.9 | | | | |
| FGF10 | 2 51.7 | 5 44.3 | Z 14.0 | FGF7 | 3 39.3 | 15 49.4 | 10 10.5 | FGF22 | 19 7.7 | 19 0.6 | 28 2.7 | FGF3 | 27 10.9 | 11 69.8 | 5 17.6 |
| SYT10 | 8 3.4 | 12 33.4 | 1 58.7 | SYT6 | 3 28.8 | 1 114.1 | 26 3.8 | SYT3 | - | 19 50.6 | - | SYT9 | 27 8.9 | 11 7.2 | 5 7.2 |
| PKP2 | 8 3.2 | 12 32.8 | 1 58.9 | PKP1 | 3 24.1 | 1 201.3 | 26 0.8 | – | | | | PKP3 | 27 14.6 | 11 0.4 | 5 1.5 |
| CYB5R3 | 8 3.7 | 22 42.6 | 1 68.4 | CYB5R1 | 3 26.7 | 1 203.0 | - | – | | | | CYB5R2 | 27 8.8 | 11 7.7 | 5 7.4 |
| PACSIN2 | 8 1.5 | 22 42.8 | 1 68.5 | PACSIN1 | 3 25.9 | 6 34.5 | 26 4.4 | – | | | | PACSIN3 | 27 11.7 | 11 47.2 | 5 23.2 |
| TEAD4 | 8 41.6 | 12 3.0 | 1 75.5 | TEAD3 | 3 23.4 | 6 35.5 | 26 38.4 | TEAD2 | - | 19 49.3 | - | TEAD1 | 27 8.4 | 11 12.7 | 5 7.7 |
| KCNC2 | 8 28.7 | 12 75.0 | 1 37.6 | KCNC4 | 3 33.6 | 1 110.2 | 26 1.3 | KCNC3 | ? | 19 50.3 | - | KCNC1 | 27 14.7 | 11 17.7 | 5 12.2 |
| SLC5A8 | 8 2.8 | 12 101.2 | 1 47.6 | SLC5A8L | 3 29.5 | - | - | SLC5A5 | 19 4.4 | 19 17.9 | 28 3.3 | SLC5A12 | 27 13.8 | 11 26.7 | 5 3.4 |
| SLC17A8 | 8 3.1 | 12 100.4 | 1 47.3 | – | | | | SLC17A7 | ? | 19 49.4 | - | SLC17A6 | 27 14.4 | 11 22.3 | 5 2.8 |
| **GCIP** | 8 3.3 | - | - | | | | | | | | | | | | |
| | | | | **GUCA1B** | 3 32.7 | 6 42.2 | 26 3.2 | | | | | **GCAP2A**<br>**GCAP2B** | 27 11.4 | - | - |
| **GCAP1–L** | 8 8.2 | - | 1 9.9<br>20.9 | **GUCA1A** | 3 32.7 | 6 42.2 | 26 3.2 | **GUCA1C** | 3 18.6 | 3 108.9 | 1 87.7 | | | | |

| | | | | | | | | | | | | | | | | | | | | | | | | | | | |
|---|---|---|---|---|---|---|---|---|---|---|---|---|---|---|---|---|---|---|---|---|---|---|---|---|---|---|---|
| LHFPL3 | 8 | 8.4 | 7 | 104.3 | 1 | 13.5 | LHFPL5 | 3 | 32.3 | 6 | 35.8 | 26 | 93.8 | LHFPL4B | 1 | 2.8 | - | | - | | LHFPL4 | 5 | 44.5 | 3 | 9.5 | 12 | 11.4 |
| SRPK2 | 8 | 8.5 | 7 | 105.1 | 1 | 13.8 | SRPK1 | 3 | 32.3 | 6 | 35.8 | 26 | 0.1 | SRPK3 | 1 | 2.3 | X | 153.8 | - | | SRPK3L | 5 | 44.4 | - | | 12 | 11.4 |
| PLXNB2 | 8 | 8.0 | 22 | 50.3 | 1 | 20.5 | – | | | | | | | PLXNB3 | 1 | 2.3 | X | 153.8 | - | | PLXNB1 | 5 | 44.4 | 3 | 48.4 | 12 | 11.3 |
| PLXNA4 | 8 | 11.9 | 7 | 132.1 | 1 | 2.3 | PLXNA2 | 3 | 30.7 | 1 | 208.0 | 26 | 2.8 | PLXNA3 | 1 | 1.9 | X | 154.5 | - | | PLXNA1 | 5 | 45.3 | 3 | 127.0 | 12 | 9.9 |
| **SWS1** | 8 | 10.4 | 7 | 128.8 | ? | | **RH2** | 3 | 36.2 | - | | 26 | 4.6 | **SWS2** | 1 | 2.6 | - | | ? | | **RH1** | 5 | 23.7 | 3 | 129.9 | 12 | 19.6 |
| | | | | | | | | | | | | | | **LWS** | 1 | 2.6 | X | 154.1 | ? | | | | | | | | |
| GNAI1 | 8 | 21.7 | 7 | 80.1 | 1 | 11.4 | GNAI3 | 3 | 36.3 | 1 | 109.5 | 26 | 1.2 | – | | | | | | | GNAI2 | 5 | 26.8 | 3 | 50.2 | 12 | 3.2 |
| GNAT3 | - | | 7 | 80.5 | 1 | 11.3 | **GNAT2** | 3 | 36.3 | 1 | 109.6 | 26 | 1.2 | – | | | | | | | **GNAT1** | 5 | 26.8 | 3 | 50.2 | 12 | 3.2 |
| KCND2 | 8 | 16.0 | 7 | 120.3 | 1 | 23.8 | KCND3 | 3 | 28.2 | 1 | 111.8 | 26 | 3.3 | KCND1 | 1 | 1.2 | X | 49.0 | - | | – | | | | | | |
| GRM8 | 8 | 17.0 | 7 | 126.4 | 1 | 20.9 | GRM4 | 3 | 25.8 | 6 | 34.0 | 26 | 4.4 | GRM6 | 1 | 1.5 | 5 | 179.0 | - | | GRM7 | 5 | 42.1 | 3 | 6.8 | 12 | 18.9 |

**Supplementary Table S2**. Gene synteny in the vicinity of GCAP3 (*GUCA1C*).

Columns 'LG', 'Hsa', 'Mmu', 'Mdo', 'Gga', 'Aca', 'Xtr' give chromosome numbers.
Columns 'Gar', 'Human', 'Mouse', 'Opossum', 'Chicken', 'Anole', 'Xenopus' give gene location in megabases.
'–' denotes gene missing from this taxon; GL numbers are unplaced scaffolds.

| Protein | Gene | LG | Gar | Hsa | Human | Mmu | Mouse | Mdo | Opossum | Gga | Chicken | Aca | Anole | Xtr | Xenopus |
|---|---|---|---|---|---|---|---|---|---|---|---|---|---|---|---|
| | CD47 | 3 | 18.7 | 3 | 108.0 | 16 | 49.8 | 4 | 67.4 | 1 | 87.4 | 3 | 169.6 | – | |
| | TRAT1 | 3 | 18.6 | 3 | 108.8 | 16 | 48.7 | 4 | 68.8 | 1 | 87.7 | – | | GL172659 | 1.1 |
| GCAP3 | GUCA1C | 3 | 18.6 | 3 | 108.9 | – | | 4 | 69.0 | 1 | 87.7 | 3 | 169.0 | GL173200 | 0.7 |
| | NECTIN3 | 3 | 19.1 | 3 | 111.1 | 16 | 46.4 | 4 | 71.8 | 1 | 88.2 | 3 | 168.2 | GL173200 | 0.4 |
| | C3orf52 | 3 | 18.7 | 3 | 112.1 | 16 | 45.6 | 4 | 73.4 | 1 | 88.7 | 3 | 167.6 | – | |
| | TAGLN3 | 3 | 5.8 | 3 | 112.0 | 16 | 45.7 | 4 | 73.2 | 1 | 88.6 | 3 | 167.7 | – | |
| | TMPRSS7 | 3 | 5.8 | 3 | 112.0 | 16 | 45.7 | 4 | 73.2 | 1 | 88.6 | 3 | 167.7 | – | |
| GCAP1 | GUCA1A | 3 | 32.7 | 6 | 42.2 | 17 | 47.4 | 2 | 285.3 | 26 | 3.2 | 4 | 126.4 | GL172697 | 1.5 |
| GCAP2 | GUCA1B | 3 | 32.7 | 6 | 42.2 | 17 | 47.4 | 2 | 285.3 | 26 | 3.2 | 4 | 126.4 | GL172697 | 1.5 |

**Supplementary Table S3**. Summary of transcripts examined.

| # | Comp. | Label on Figures | Accession number | aa's | Join | M | * | RPKM | RPKM -CDS | Foot-note |
|---|---|---|---|---|---|---|---|---|---|---|
| 1 | RecVis-X | RecVis-X G.australis 57785-5-2 | MH577347 | 193 | | M | * | 979.8 | 1640.1 | |
| 2 | RecVis-X | RecVis-X M.mordax 42083-4-2 | MH577348 | 193 | | M | * | 327.9 | 433.8 | |
| 3 | RecVis-Y | RecVis-Y G.australis 48614-1-1 | MH577349 | 195 | | M | * | 27.5 | 34.7 | |
| 4 | RecVis-Y | RecVis-Y M.mordax 24130-1-1 | MH577350 | 195 | | M | * | 44.3 | 68.0 | |
| 5 | Recoverin | Recoverin Western ray 15444-1-2 | MH577351 | 200 | | M | * | 663.6 | 1094.8 | |
| 6 | Recoverin | Recoverin Bluespot ray 28481-1-2 | MH577352 | 200 | | M | * | 1337.0 | 2163.0 | |
| 7 | Recoverin | Recoverin Bamboo shark 52692-1-1 | MH577353 | 200 | | M | * | 722.8 | 1071.2 | |
| 8 | Recoverin | Recoverin Bowfin 56843-1-1 | MH577354 | 202 | | M | * | 820.5 | 1700.9 | |
| 9 | Recoverin | Recoverin Florida gar 20327-1-2 | MH577355 | 202 | | M | * | 1019.2 | 1260.8 | |
| 10 | Visinin | Visinin Bowfin 14041-1-1 | MH577356 | 194 | | M | * | 730.4 | 903.5 | |
| 11 | Visinin | Visinin Florida gar 26621-3-1 | MH577357 | 194 | | M | * | 510.6 | 384.0 | |
| 12 | GCAP | [ GCAP E.cirrhatus 145347-1-3w144238-2-1 ] | MH577358 | 197 | J | M | * | 289.8 | 471.4 | 1 |
| 13 | GCAP1-X | GCAP1-X G.australis 59152-11-2 | MH577359 | 189 | | M | * | 162.9 | 252.0 | |
| 14 | GCAP1-X | GCAP1-X M.mordax 38287-3-2 | MH577360 | 189 | | M | * | 58.3 | 85.9 | |
| 15 | GCAP1-Y | GCAP1-Y G.australis 51338-1-1 | MH577361 | 188 | | M | * | 4.9 | 5.8 | |
| 16 | GCAP1-L | GCAP1-L Western ray 41716-3-1 | MH577362 | 188 | | M | * | 132.7 | 143.3 | |
| 17 | GCAP1-L | GCAP1-L Bluespot ray 28739-1-1 | MH577363 | 188 | | M | * | 219.5 | 310.4 | |
| 18 | GCAP1-L | GCAP1-L Reef shark 54755-8-1 | MH577364 | 188 | | M | * | 400.6 | 468.1 | |
| 19 | GCAP1-L | GCAP1-L Bamboo shark 38561-2-1 | MH577365 | 188 | | M | * | 72.6 | 83.9 | |
| 20 | GCAP1-L | GCAP1-L Bowfin 29798-1-1 | MH577366 | 188 | | M | * | 262.6 | 336.5 | |
| 21 | GCAP1-L | GCAP1-L Florida gar 23848-2-1w1-1 | MH577367 | 188 | J | M | * | 254.9 | 238.6 | |
| 22 | GCAP1 | GCAP1 Bowfin 19776-1-1 | MH577368 | 194 | | M | * | 169.3 | 176.5 | |
| 23 | GCAP1 | GCAP1 Florida gar 24484-1-1 | MH577369 | 195 | | M | * | 305.1 | 273.4 | |
| 24 | GCAP3 | GCAP3 Bowfin 27277-1-3 | MH577370 | 187 | | M | * | 66.1 | 57.1 | |
| 25 | GCAP3 | GCAP3 Florida gar 29324-1-1 | MH577371 | 187 | | M | * | 38.9 | 40.6 | |
| 26 | GCAP2 | GCAP2 G.australis 57165-1-2 | MH577372 | 201 | | M | * | 197.0 | 299.1 | |
| 27 | GCAP2 | GCAP2 M.mordax 38980-1-1 | MH577373 | 201 | | M | * | 136.3 | 181.0 | |
| 28 | GCAP2 | GCAP2 Western ray 37932-1-1 | MH577374 | 196 | | M | * | 377.5 | 597.2 | |
| 29 | GCAP2 | GCAP2 Bluespot ray 41809-1-1 | MH577375 | 196 | | M | * | 944.5 | 1471.6 | |
| 30 | GCAP2 | GCAP2 Reef shark 49130-1-1 | MH577376 | 196 | | M | * | 848.9 | 1506.5 | |
| 31 | GCAP2 | GCAP2 Bamboo shark 45556-1-1 | MH577377 | 196 | | M | * | 237.7 | 351.0 | |
| 32 | GCAP2 | GCAP2 Bowfin 22440-1-1 | MH577378 | 197 | | M | * | 499.4 | 420.9 | |
| 33 | GCAP2 | GCAP2 Florida gar 28304-1-1 | MH577379 | 197 | | M | * | 206.5 | 116.9 | |
| 34 | GCAP2-A | GCAP2-A Bowfin 45476-1-1 | MH577380 | 196 | | M | * | 58.8 | 71.2 | |
| 35 | GCAP2-A | GCAP2-A Florida gar 16213-1-1 | MH577381 | 196 | | M | * | 75.9 | 182.1 | |
| 36 | GCAP2-B | GCAP2-B Bowfin 21214-1-2 | MH577382 | 192 | | M | * | 21.7 | 36.3 | |
| 37 | GCAP2-B | GCAP2-B Florida gar 22853-1-2 | MH577383 | 192 | | M | * | 39.7 | 46.8 | |
| 38 | GC-Y | GC-Y E.cirrhatus 138078-2-1 | MH577384 | 1087 | | M | * | 15.3 | 16.3 | |
| 39 | GC-X | GC-X G.australis 42895-1-1 | MH577385 | 1070 | | | * | 9.8 | 11.2 | |
| 40 | GC-X | GC-X M.mordax 41168-13-1 | MH577386 | 1053 | | | * | 23.0 | 22.4 | |
| 41 | GC-E | GC-E Western ray 40955-2-1 | MH577387 | 1114 | | M | * | 9.9 | 10.3 | |
| 42 | GC-E | GC-E Bluespot ray 37339-2-1 | MH577388 | 1138 | | M | * | 28.1 | 32.7 | |
| 43 | GC-E | GC-E-Partial Bamboo shark 32459-1-1 | MH577389 | 806 | | | | 1.8 | 1.8 | |
| 44 | GC-E | GC-E-Partial Bowfin 26069-7-2w1-2 | MH577390 | 657 | J | | * | 5.1 | 4.8 | |
| 45 | GC-F | GC-F Western ray 41527-1-3 | MH577391 | 1129 | | M | * | 28.2 | 32.6 | |

| | | | | | | | | | | |
|---|---|---|---|---|---|---|---|---|---|---|
| 46 | GC-F | GC-F Bluespot ray 36824-9-2 | MH577392 | 1129 | | M | * | 90.9 | 87.7 | |
| 47 | GC-F | GC-F Reef shark 51923-1-1 | MH577393 | 1111 | | M | * | 84.4 | 102.1 | |
| 48 | GC-F | GC-F Bamboo shark 43879-1-2 | MH577394 | 1147 | | M | * | 39.5 | 43.1 | |
| 49 | GC-F | [ GC-F Florida gar 29955-2-2 ] | MH577395 | 1110 | | M | * | 45.5 | 21.1 | 2 |
| 50 | GC-D | GC-D Western ray 41905-4-3 | MH577396 | 1109 | | M | * | 58.9 | 63.8 | |
| 51 | GC-D | GC-D Bluespot ray 37091-10-1 | MH577397 | 1109 | | M | * | 38.7 | 47.1 | |
| 52 | GC-D | GC-D Reef shark 55268-1-3 | MH577398 | 1109 | | M | * | 56.8 | 63.1 | |
| 53 | GC-D | GC-D Bamboo shark 45560-9-1 | MH577399 | 1109 | | M | * | 92.4 | 96.7 | |
| 54 | GC-D | GC-D Bowfin 27177-5-1w4-2 | MH577400 | 1114 | J | M | * | 15.7 | 17.5 | |
| 55 | GC-D | GC-D Florida gar 29955-2-3 | MH577401 | 1109 | | M | * | 44.0 | 20.3 | |
| 56 | NCKX | NCKX E.cirrhatus 143862-1-3-FS | MH577402 | 689 | | M | * | 2.3 | 1.8 | FS |
| 57 | NCKX | [ NCKX-Partial E.cirrhatus 144620-12-1 ] | MH577403 | 332 | | | * | 9.5 | 10.8 | 3 |
| 58 | NCKX-X | NCKX-X-Partial G.australis 57547-1-1w5-1 | MH577404 | 204 | J | | * | 3.0 | 1.1 | |
| 59 | NCKX-Y | NCKX-Y-Non-contig G.australis 39322-2-1w 57547-2-1 | MH577405 | 465 | NJ | | * | 0.9 | 1.0 | |
| 60 | NCKX2 | NCKX2 G.australis 56087-6-2 | MH577406 | 673 | | M | * | 13.6 | 15.4 | |
| 61 | NCKX2 | NCKX2-Partial M.mordax 39865-1-2 | MH577407 | 571 | | | * | 19.3 | 20.4 | |
| 62 | NCKX2 | NCKX2 Western ray 40662-2-5 | MH577408 | 675 | | M | * | 16.2 | 14.6 | |
| 63 | NCKX2 | NCKX2 Bluespot ray 35629-1-1 | MH577409 | 646 | | M | * | 17.6 | 11.9 | |
| 64 | NCKX2 | NCKX2-Partial Reef shark 46003-2-1w3-2 | MH577410 | 587 | J | | * | 2.9 | 2.9 | |
| 65 | NCKX2 | NCKX2 Bamboo shark 43754-1-1 | MH577411 | 654 | | M | * | 6.5 | 6.6 | |
| 66 | NCKX2 | NCKX2 Bowfin 26993-6-1w2-3 | MH577412 | 645 | J | M | * | 48.4 | 22.1 | |
| 67 | NCKX2 | NCKX2 Florida gar 27907-1-2 | MH577413 | 661 | | M | * | 23.1 | 8.0 | |
| 68 | NCKX1 | NCKX1 Western ray 41546-5-1 | MH577414 | 762 | | M | * | 79.3 | 108.4 | |
| 69 | NCKX1 | NCKX1 Bluespot ray 36644-8-4 | MH577415 | 848 | | M | * | 80.9 | 85.9 | |
| 70 | NCKX1 | NCKX1 Reef shark 55666-1-2 | MH577416 | 842 | | M | * | 203.8 | 199.4 | |
| 71 | NCKX1 | NCKX1-Partial Bamboo shark 38438-1-4 | MH577417 | 733 | | | * | 98.0 | 119.7 | |
| 72 | NCKX1 | NCKX1 Bowfin 24833-1-2 | MH577418 | 703 | | M | * | 26.0 | 21.9 | |
| 73 | NCKX1 | [ NCKX1-Partial Florida gar 25860-2-2 ] | MH577419 | 590 | | | * | 30.8 | – | 4 |

**Explanation of columns**

Column 3, 'Label on Figures': Sequences are indicated '-Partial' if they included fewer than 95% of the expected residues. The four entries in square brackets do not appear in phylogenies for reasons set out in the footnotes below.

Column 6, 'Join': 'J' indicates the sequence was manually joined from more than one contig. Joined sequences overlapped, except where indicated 'NJ' for a non-contiguous join.

Column 7: An entry 'M' indicates that the sequence started with a Met.

Column 8. An entry '*' indicates that sequence ended with a stop codon.

Column 9: RPKM = Reads per kilobase per million mapped reads.

Column 10: RPKM-CDS = Same, but calculated across only the coding region.

**Footnotes:**

1, 3. Inclusion of the divergent hagfish sequence caused a major reduction in support for other clades in the tree.

2. The C-terminus appears dubious; the spotted gar ortholog was used for the phylogeny.

4. The full-length spotted gar ortholog was used in preference to this partial sequence. RPKM-CDS was not available due to a software bug.

FS. The sequence has been corrected for a frameshift; 39 nucleotides have been replaced by 'N's and one additional 'N' has been added.

**Supplementary Table S4**. Sequence divergence for functional domains of GCs.

| | | Human | *G. australis* | *M. mordax* |
|---|---|---|---|---|
| | *G. australis* | - | | |
| **LS** (51) | *M. mordax* | - | - | |
| | *E. cirrhatus* | 0.769 | - | - |
| | *G. australis* | 0.607 | | |
| **ECD** (411) | *M. mordax* | 0.632 | 0.155 | |
| | *E. cirrhatus* | 0.619 | 0.637 | 0.644 |
| | *G. australis* | 0.880 | | |
| **TMD** (25) | *M. mordax* | 0.760 | 0.360 | |
| | *E. cirrhatus* | 0.840 | 0.720 | 0.760 |
| | *G. australis* | 0.708 | | |
| **JMD** (116) | *M. mordax* | 0.754 | 0.224 | |
| | *E. cirrhatus* | 0.672 | 0.595 | 0.595 |
| | *G. australis* | 0.413 | | |
| **KHD** (212) | *M. mordax* | 0.417 | 0.111 | |
| | *E. cirrhatus* | 0.377 | 0.329 | 0.329 |
| | *G. australis* | 0.045 | | |
| **DD** (44) | *M. mordax* | 0.045 | 0.000 | |
| | *E. cirrhatus* | 0.045 | 0.000 | 0.000 |
| | *G. australis* | 0.101 | | |
| **CCD** (244) | *M. mordax* | 0.081 | 0.100 | |
| | *E. cirrhatus* | 0.121 | 0.212 | 0.212 |

Sequence divergence calculated across each of the seven domains of guanylyl cyclases for our three agnathan species, compared with human GC-E and with each other. The first column gives the domain name, with the number of residues in parentheses, and the values in the final three columns are the mean number of amino acid substitutions per site. The dashes in the first section indicate that divergences could not be calculated because of the large number of gaps within the N-terminal region for the sequences from the two lamprey species. The domains are defined below, with residue numbering for human GC-E.

| | | |
|---|---|---|
| LS | (1-51) | Leader sequence |
| ECD | (52-462) | Extracellular domain |
| TMD | (463-487) | Transmembrane domain |
| JMD | (488-603) | Juxta-membrane domain |
| KHD | (604-815) | Kinase homology domain |
| DD | (816-859) | Dimerization domain |
| CCD | (860-1103) | Cyclase catalytic domain |
