# Supplementary material for: Evolution of the calcium feedback steps of vertebrate phototransduction
Source: Open Biol. 2018 Sep 26;8(9):180119. doi: 10.1098/rsob.180119 (PMC6170504; doi:10.1098/rsob.180119)
Supplement: Supplementary Figures [file rsob180119supp6.pdf]

Supplementary Figures

for *Open Biology*, http://dx.doi.org/10.1098/rsob.180119

Evolution of the calcium feedback steps of vertebrate phototransduction

Trevor D. Lamb[1] and David M. Hunt[2,3]

[1]Eccles Institute of Neuroscience, John Curtin School of Medical Research, The Australian National University, ACT 2600, Australia, and [2]Centre for Ophthalmology and Visual Science, The Lions Eye Institute and [3]School of Biological Sciences, The University of Western Australia, WA 6009, Australia.

Contents

Page

Supplementary Figure S1. Unconstrained molecular phylogeny for recoverins and visinins, without outgroup. 2

Supplementary Figure S2. Fully-expanded molecular phylogeny for recoverins and visinins, corresponding to the collapsed tree in Figure 3B. 3

Supplementary Figure S3. Predicted structures for recoverins and visinins. 4

Supplementary Figure S4. Fully-expanded unconstrained molecular phylogeny for jawed vertebrate GCAPs, corresponding to the collapsed tree shown in Figure 4A. 6

Supplementary Figure S5. Constrained molecular phylogeny for GCAPs, when lamprey sequences are included, but mammalian sequences are excluded. 8

Supplementary Figure S6. Predicted structures for GCAPs. 9

Supplementary Figure S7. Fully-expanded unconstrained molecular phylogeny for jawed and agnathan vertebrate guanylyl cyclases, corresponding to the collapsed tree shown in Figure 5A. 12

Supplementary Figure S8. Molecular phylogeny for vertebrate guanylyl cyclases, with the hagfish sequence constrained to clade with the GC-E sequences. 13

Supplementary Figure S9. Functional domains for agnathan guanylyl cyclases. 14

Supplementary Figure S10. Unconstrained molecular phylogeny for NCKXs, without outgroup. 16

Supplementary Figure S11. Fully-expanded unconstrained molecular phylogeny for NCKXs, corresponding to the collapsed tree shown in Figure 6A. 17

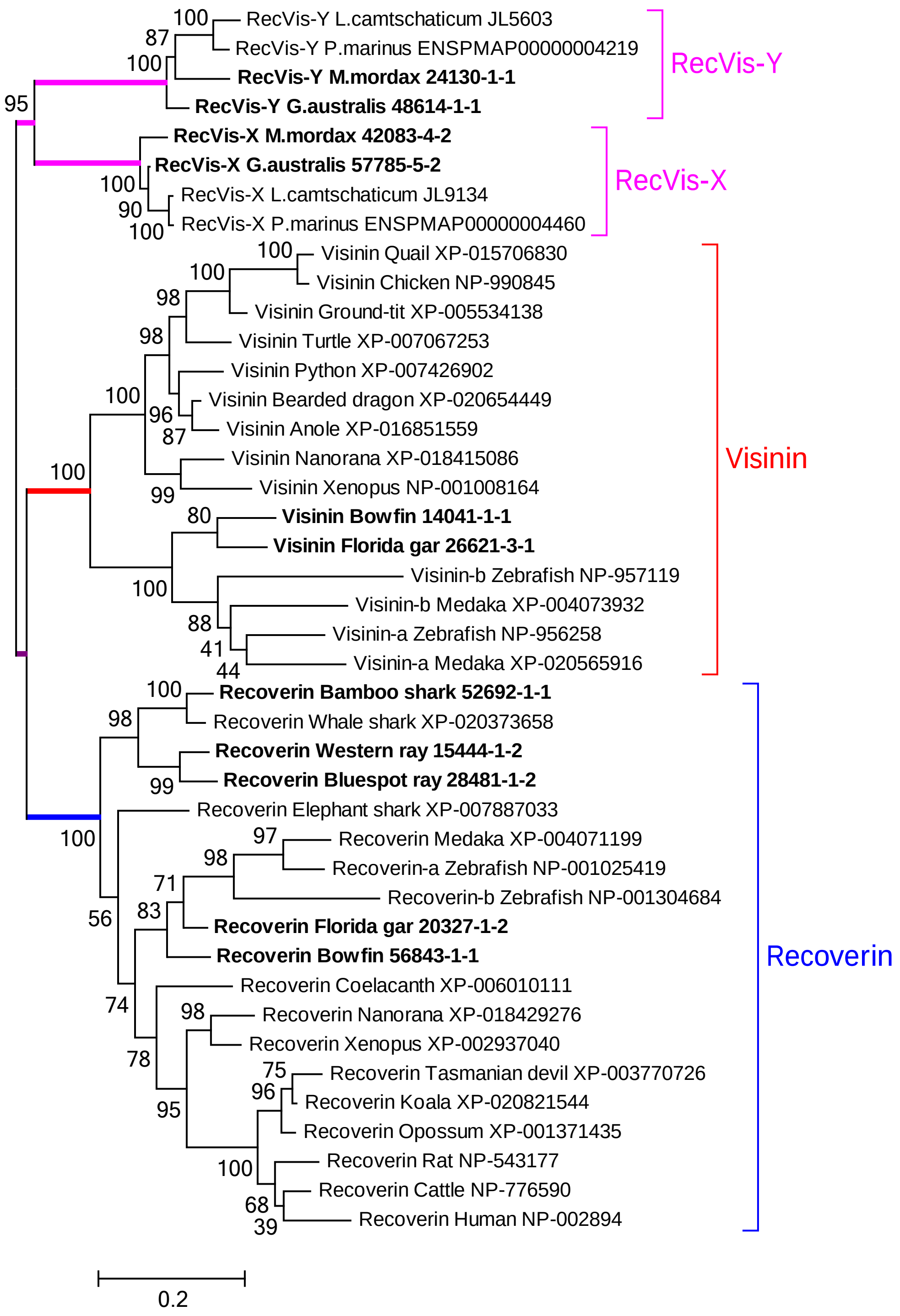

Supplementary Figure S1. Unconstrained molecular phylogeny for recoverins and visinins, without outgroup. WAG model. Bootstrap support levels for the four main sub-trees were identical using the LG model.

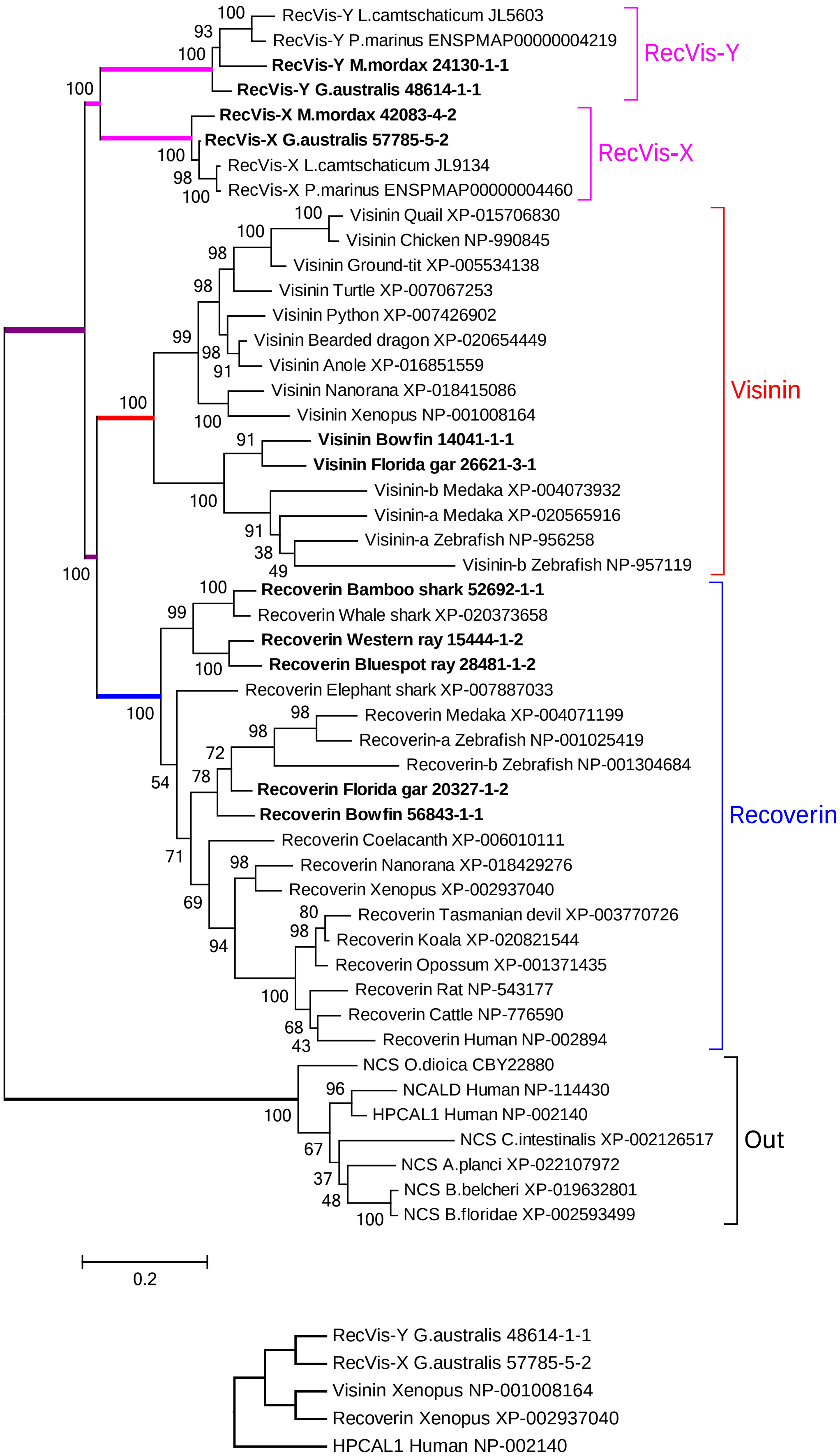

Supplementary Figure S2. Fully-expanded molecular phylogeny for recoverins and visinins, corresponding to the collapsed tree in Figure 3A. Inset shows constraint.

Supplementary Figure S3. Predicted structures for recoverins and visinins.

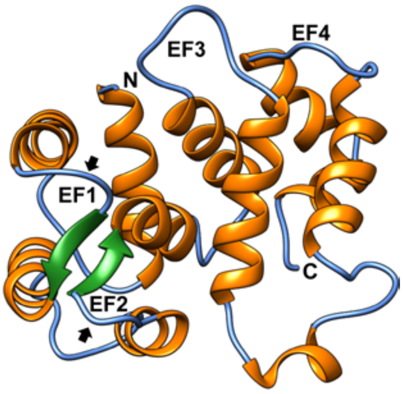

Human Recoverin

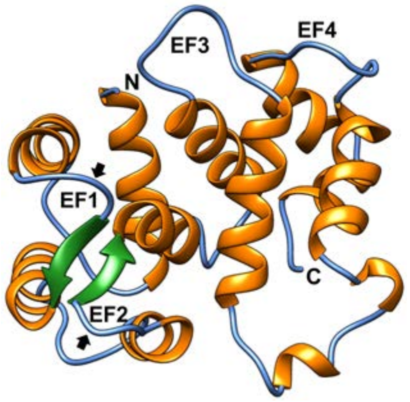

Chicken Visinin

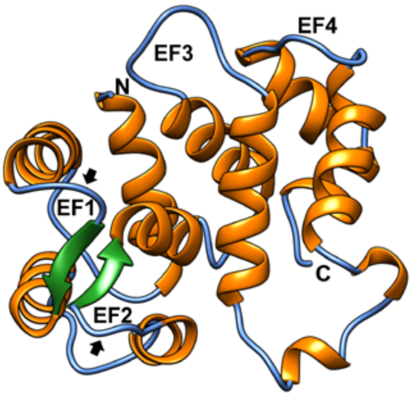

Bowfin Recoverin

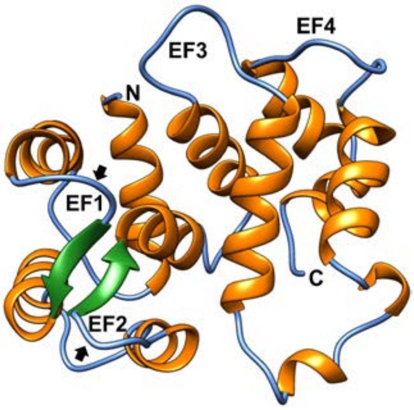

Bowfin Visinin

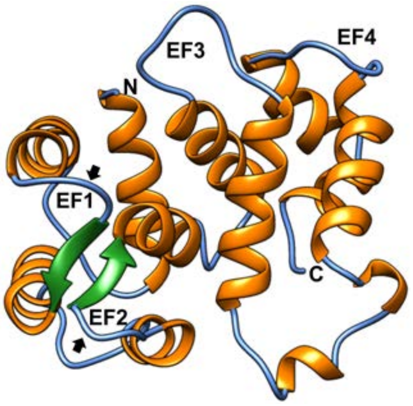

Florida gar Recoverin

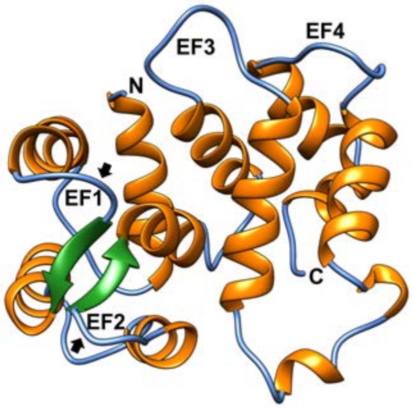

Florida gar Visinin

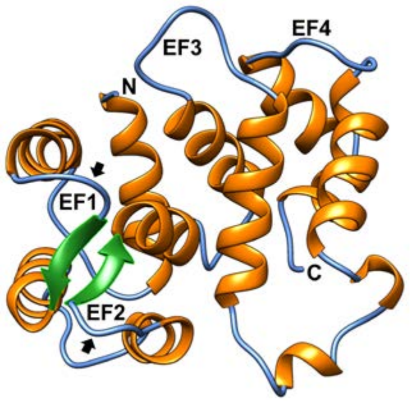

Bamboo shark Recoverin

Continued next page …

Continued from previous page…

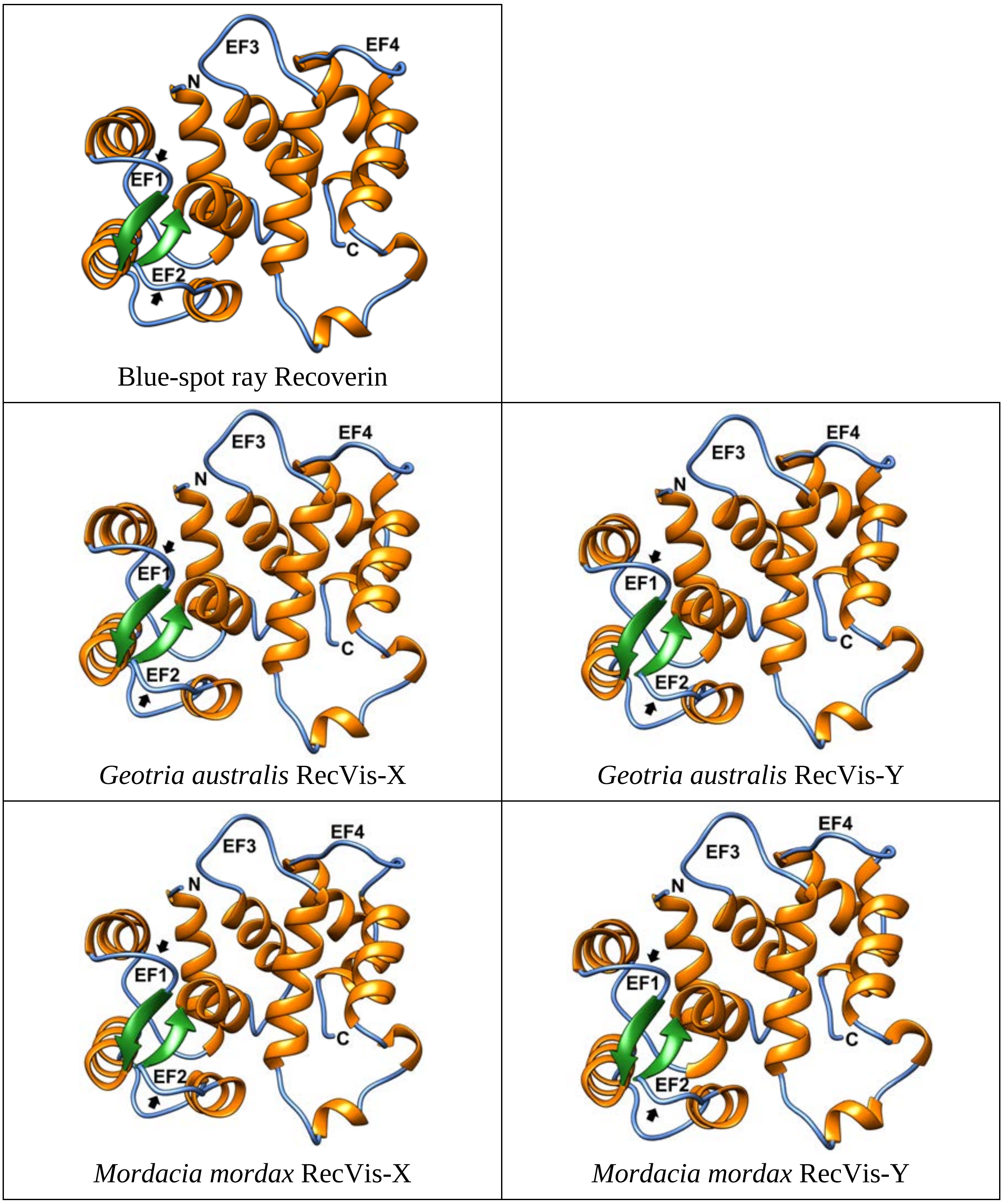

Blue-spot ray Recoverin

Geotria australis RecVis-X

Geotria australis RecVis-Y

Mordacia mordax RecVis-X

Mordacia mordax RecVis-Y

Supplementary Figure S3. Predicted structures for recoverins and visinins.

Note: Arrows are to clarify which are the EF1 and EF2 sections.

Supplementary Figure S4

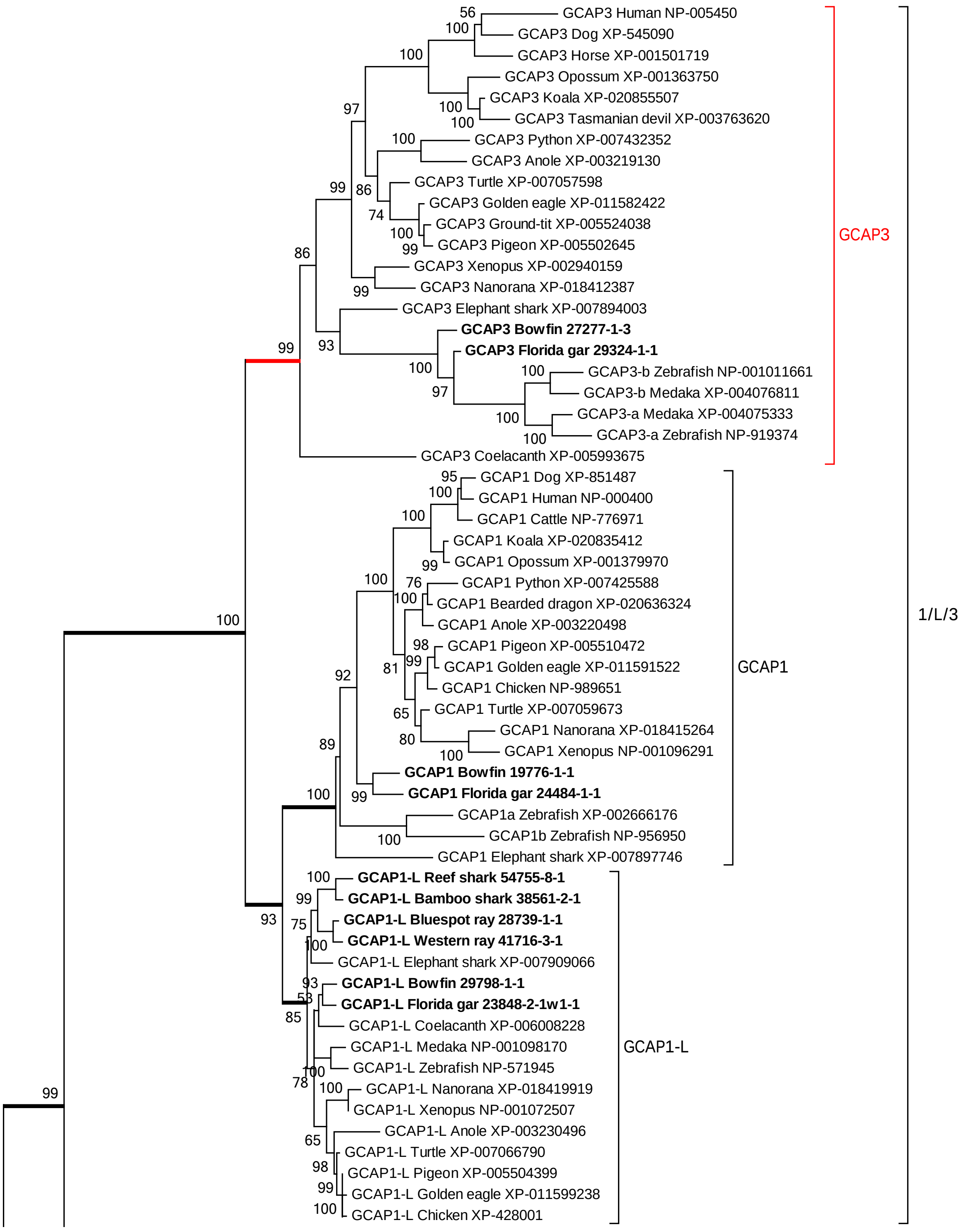

Continued …

Continued …

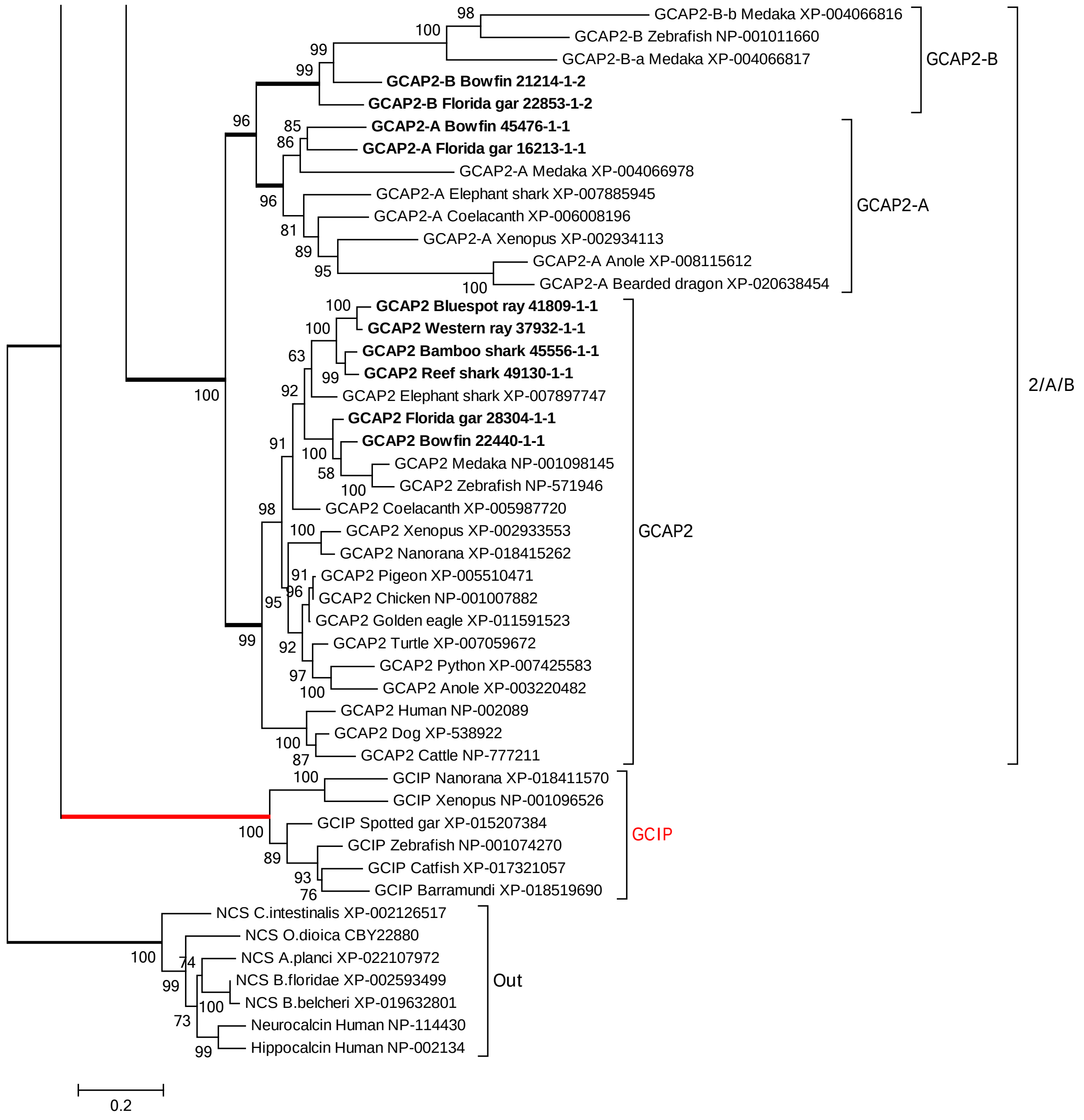

Supplementary Figure S4. Fully-expanded unconstrained molecular phylogeny for jawed vertebrate GCAPs, corresponding to the collapsed tree shown in Figure 4A.

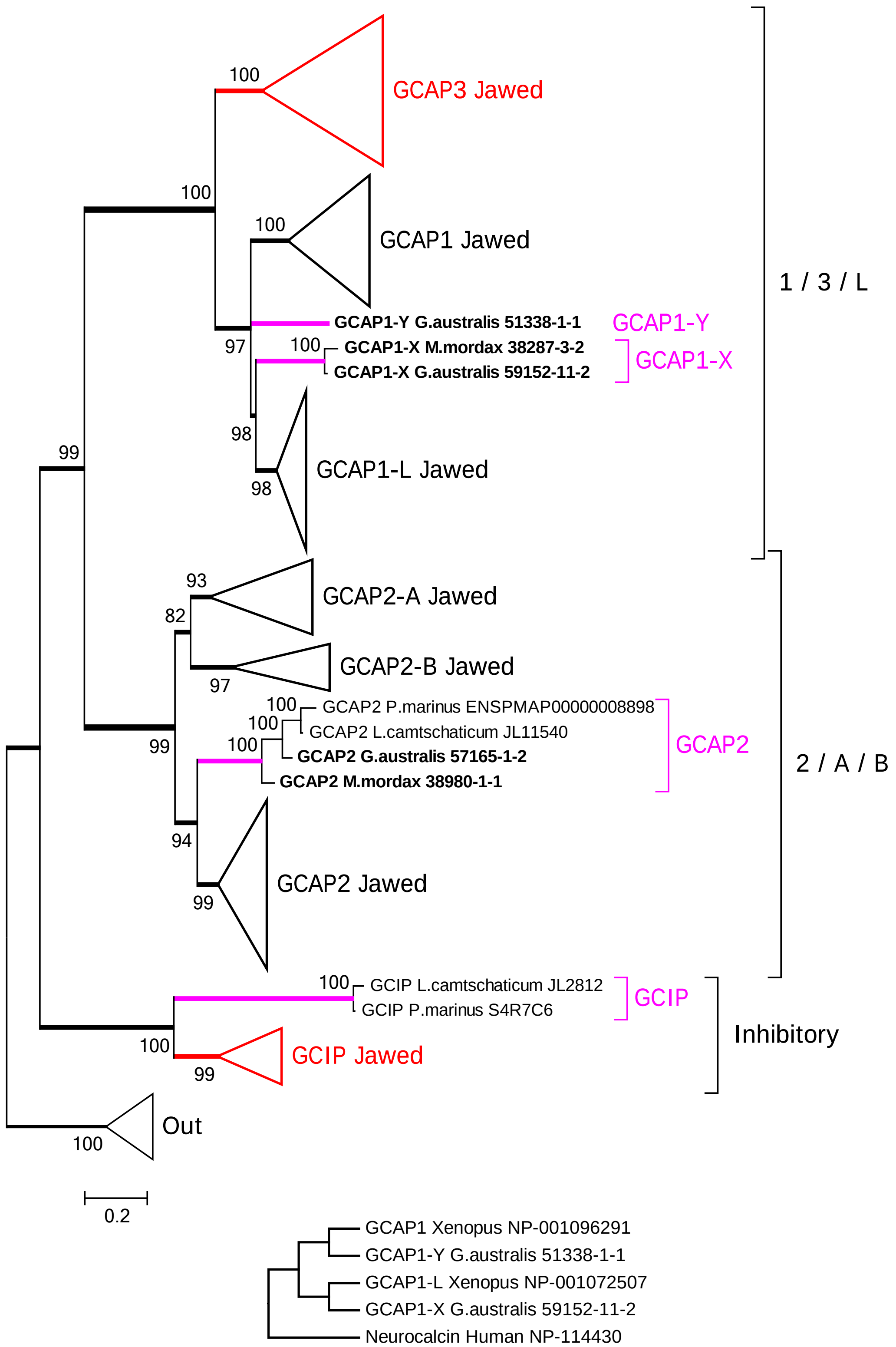

Supplementary Figure S5. Constrained molecular phylogeny for GCAPs, when lamprey sequences are included, but mammalian sequences are excluded. A highly-divergent hagfish 1/L/3 sequence was excluded, as its inclusion lowered the bootstrap support levels. The constraint tree is shown below.

Supplementary Figure S6. Predicted structures for GCAPs. **A: GCAP1** and **GCAP1-L**

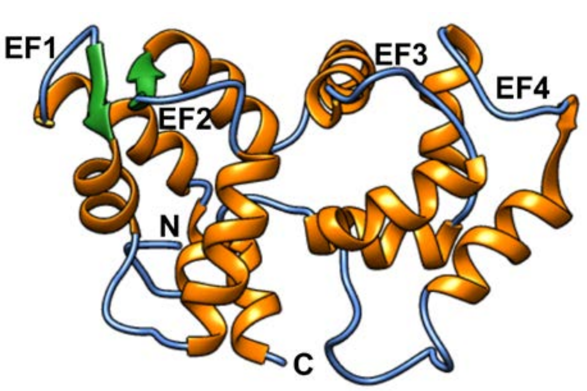

Human GCAP1

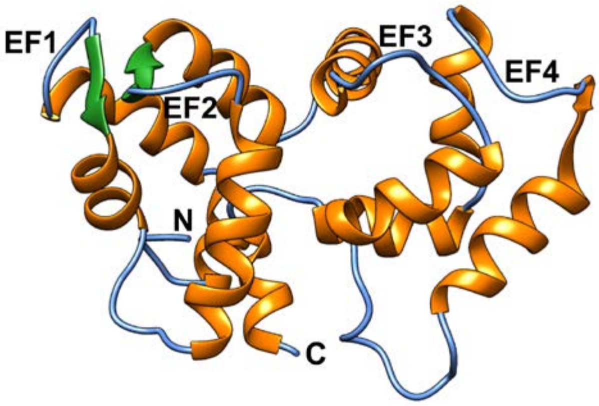

Florida gar GCAP1

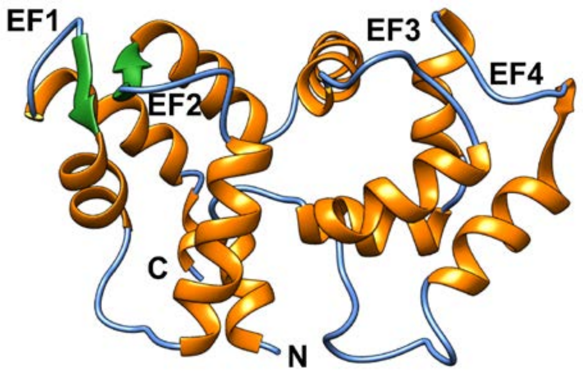

Bluespot ray GCAP1-L

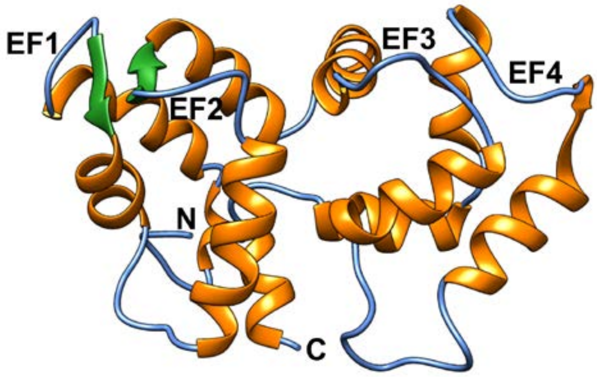

Florida gar GCAP1-L

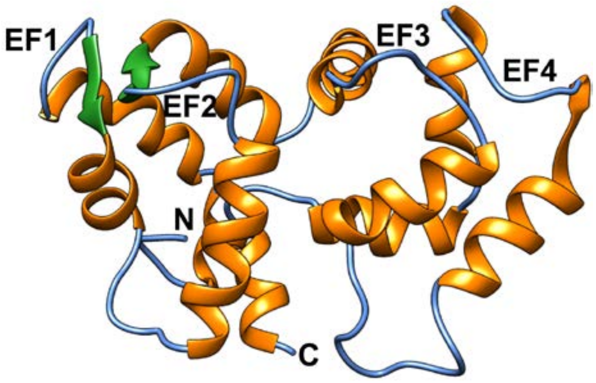

G. australis GCAP1-X

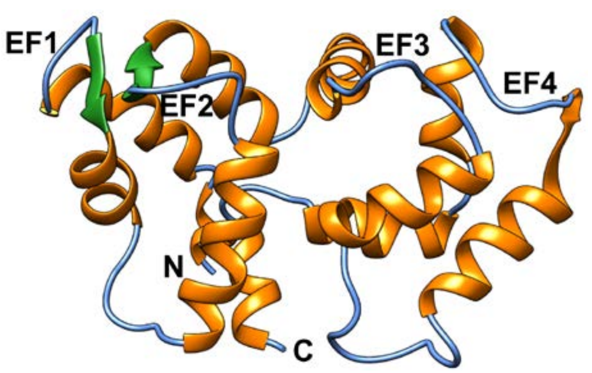

G. australis GCAP1-Y

Supplementary Figure S6. Predicted structures for GCAPs. **B: GCAP2,** and **GCAP2-A/2-B**

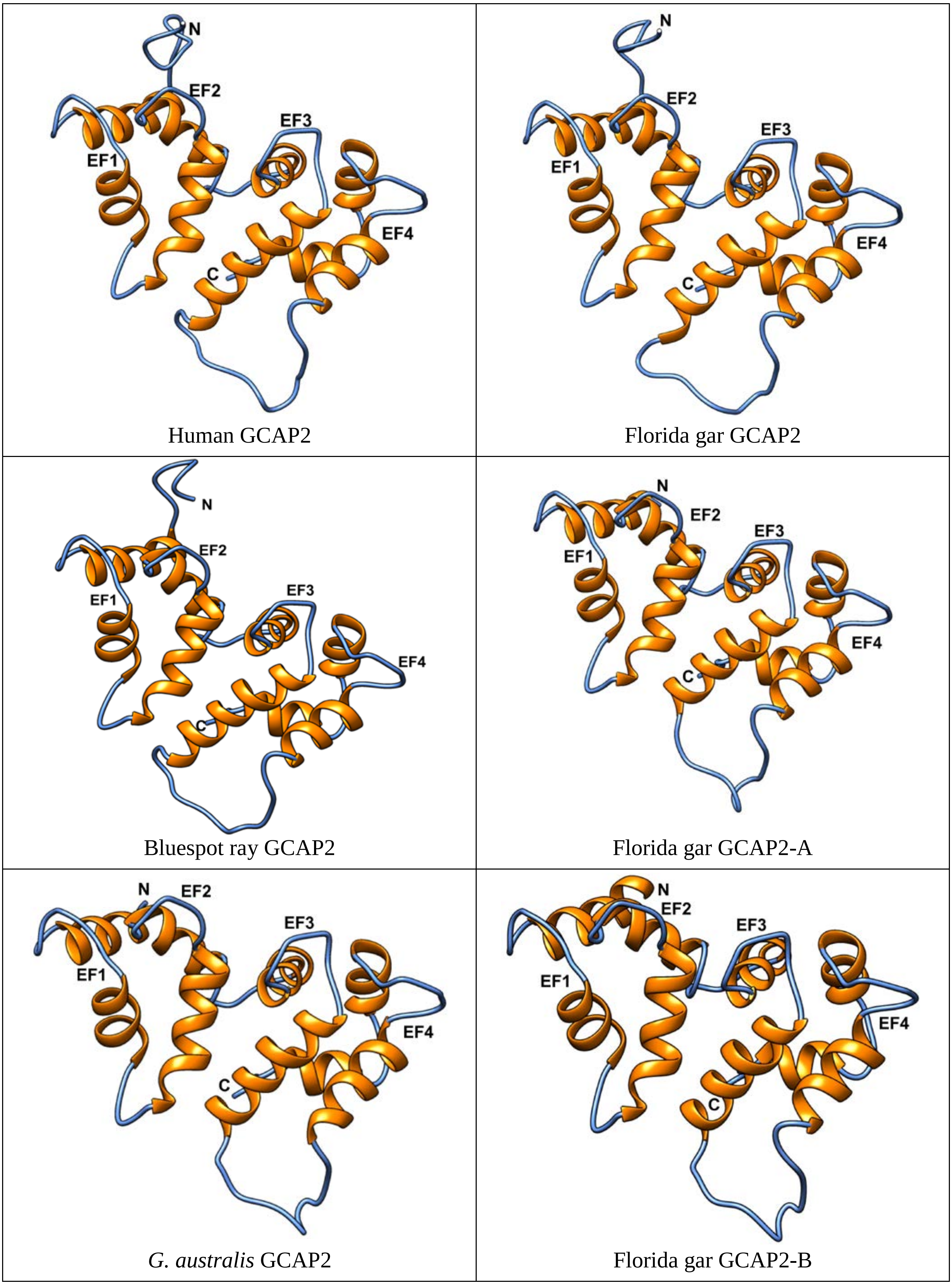

Human GCAP2 | Florida gar GCAP2

Bluespot ray GCAP2 | Florida gar GCAP2-A

G. australis GCAP2 | Florida gar GCAP2-B

Supplementary Figure S6. Predicted structures for GCAPs. **C: GCAP3** and **GCIP**

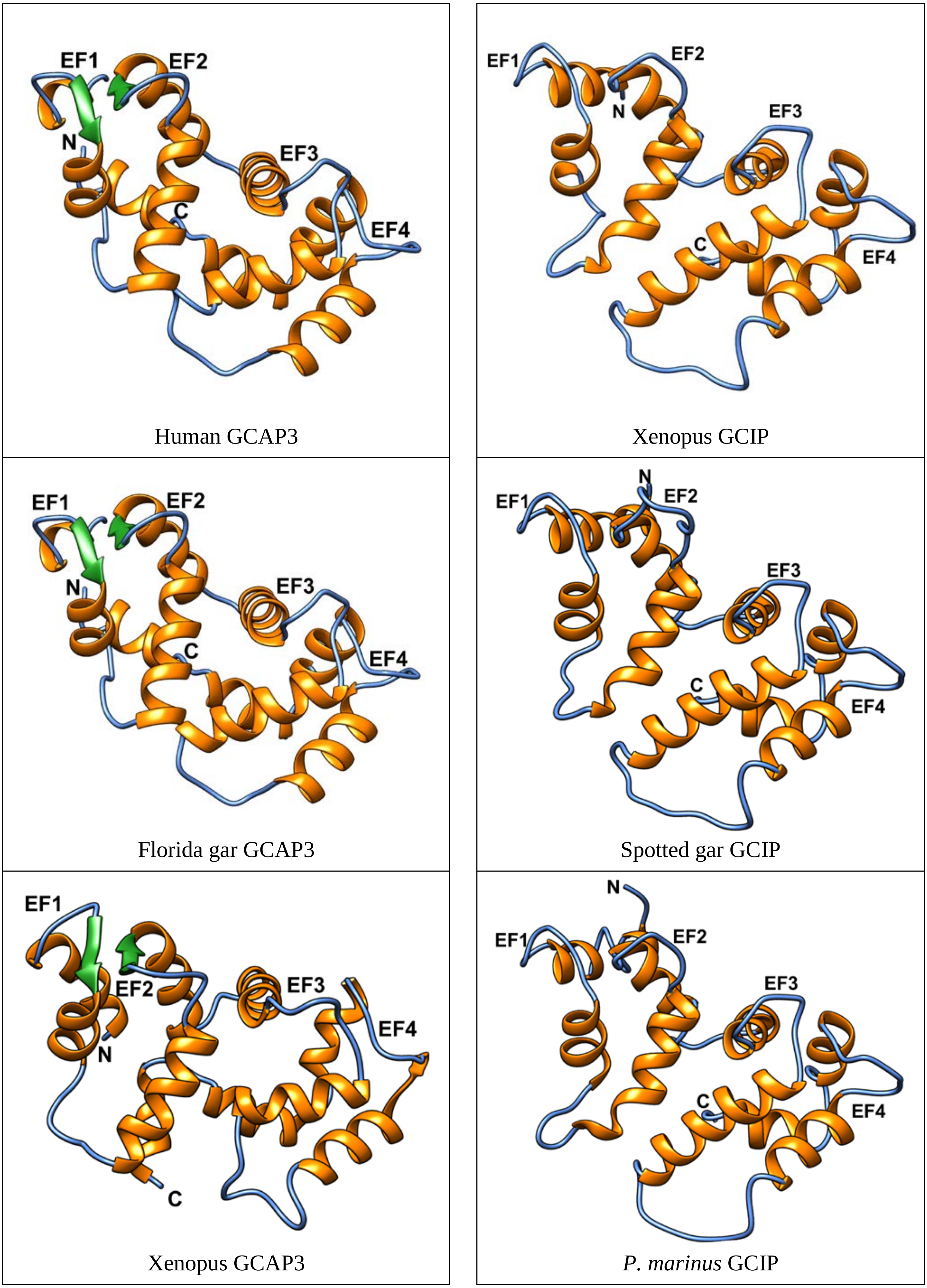

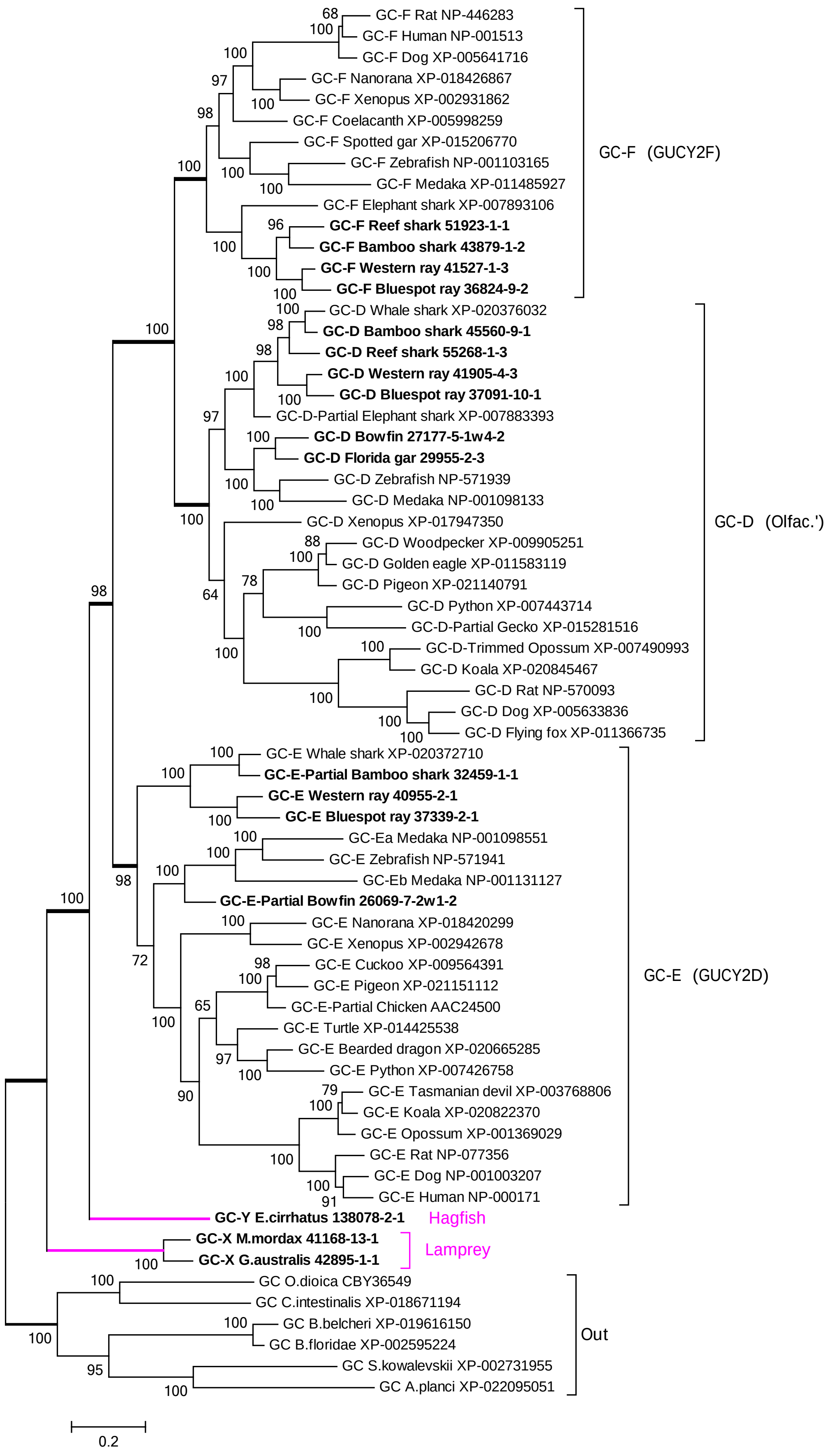

Supplementary Figure S7. Fully-expanded unconstrained molecular phylogeny for jawed and agnathan vertebrate guanylyl cyclases, corresponding to the collapsed tree shown in Figure 5A.

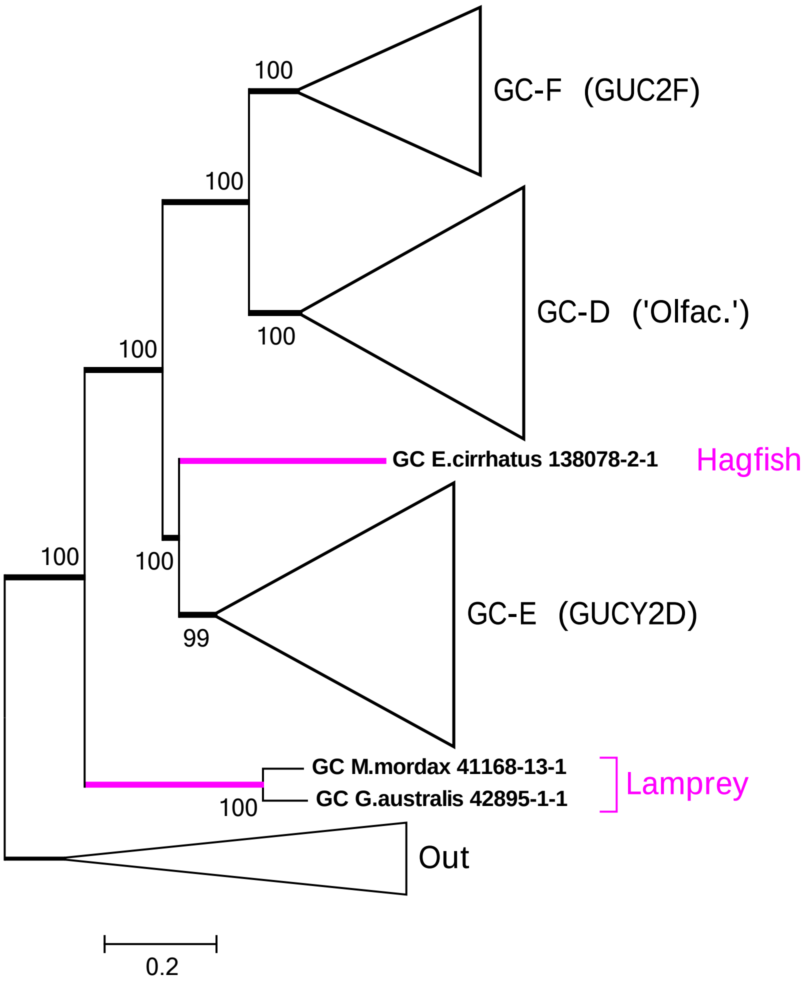

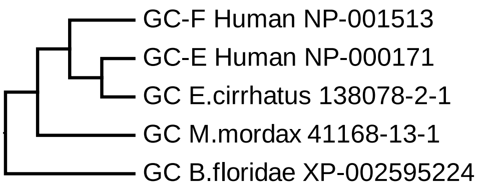

Supplementary Figure S8. Molecular phylogeny for vertebrate guanylyl cyclases, with the hagfish sequence constrained to clade with the GC-E sequences. Inset shows constraint. WAG model. The change in log likelihood from the ML tree was ΔLogL = 12.7, and the constrained tree just passed the tests of topology, with *p*-AU = 0.055. Accordingly, this tree could not be rejected.

Supplementary Figure S9. Functional domains for agnathan guanylyl cyclases.

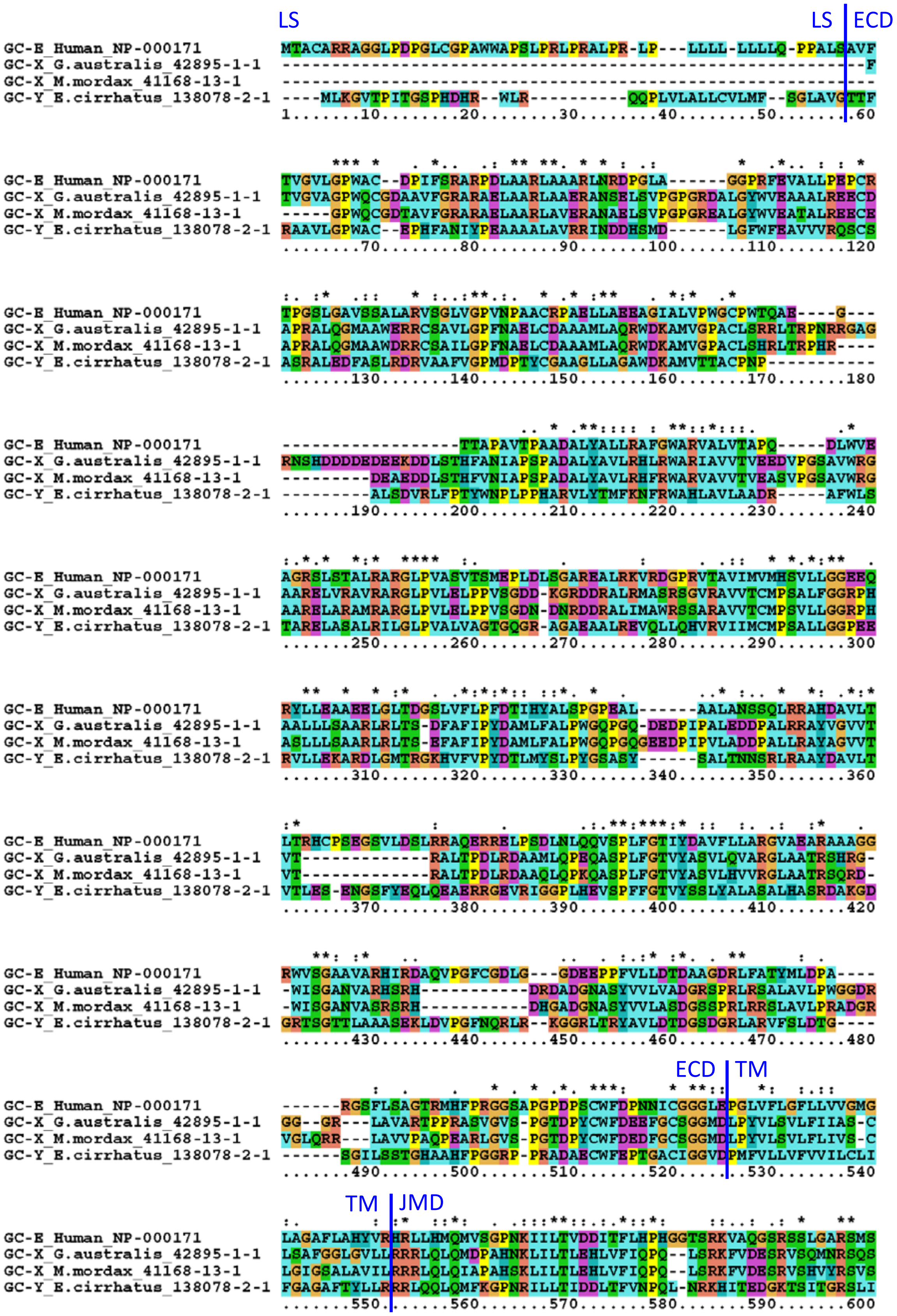

Continued …

Continued …

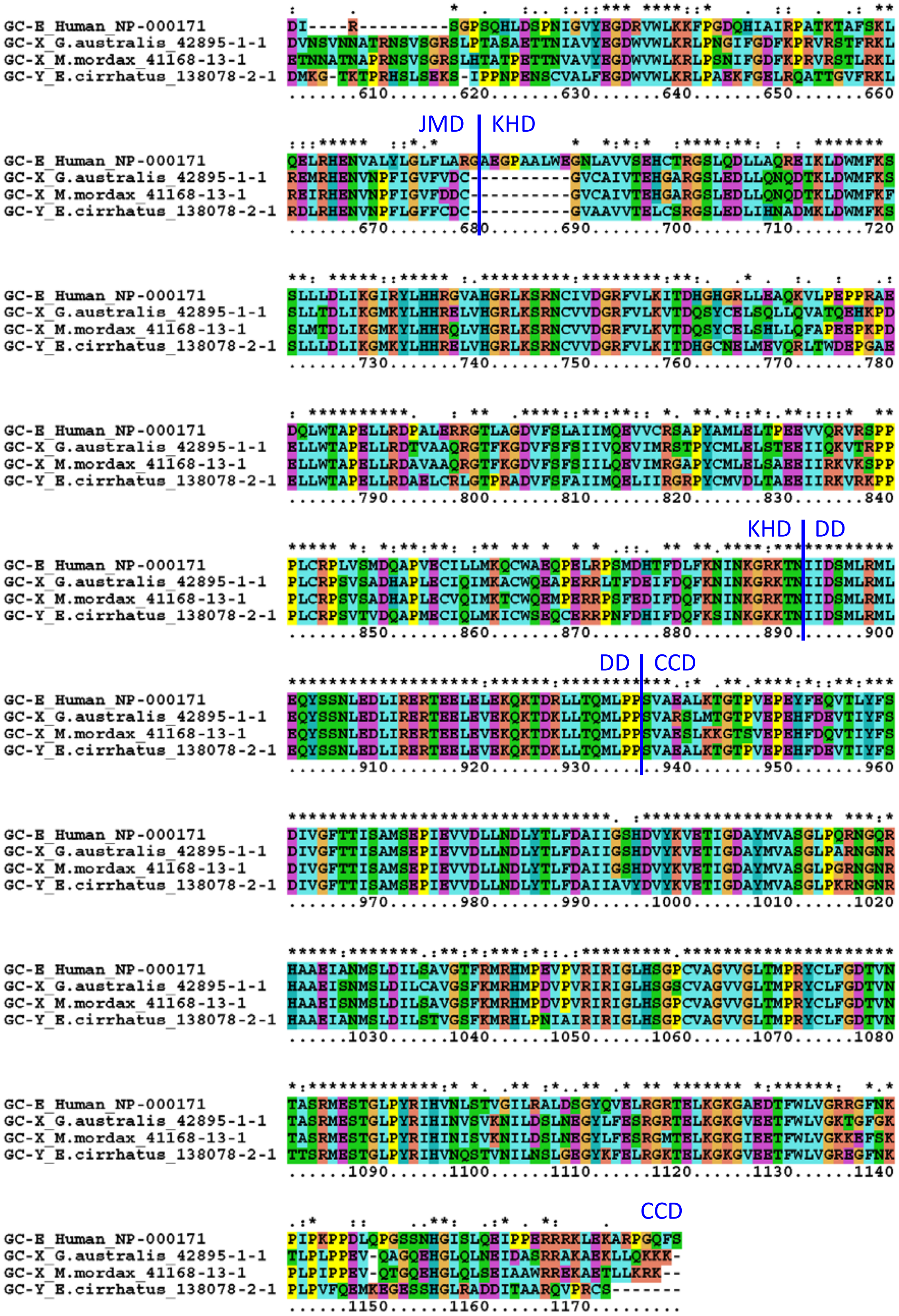

Supplementary Figure S9. Functional domains for agnathan guanylyl cyclases.

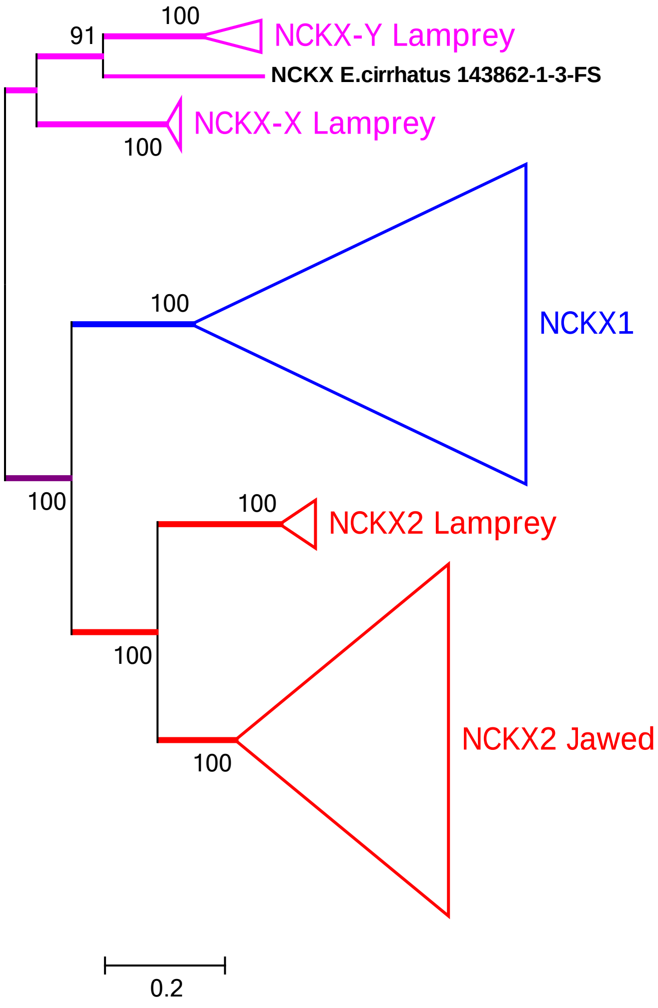

Supplementary Figure S10. Unconstrained molecular phylogeny for NCKXs, without outgroup, in collapsed form. Alignment SATé with MAFFT. WAG substitution model.

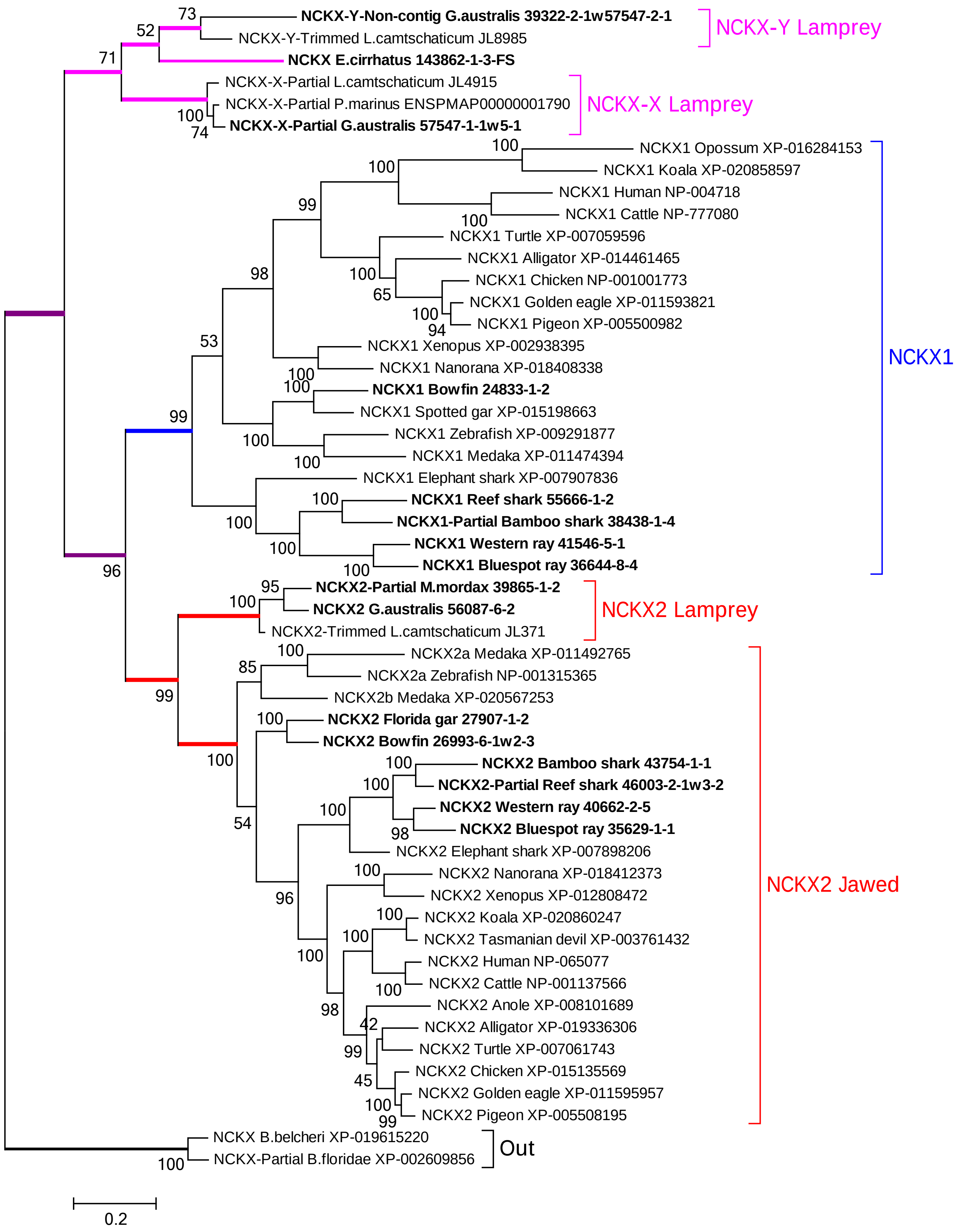

Supplementary Figure S11. Fully-expanded unconstrained molecular phylogeny for NCKXs, corresponding to the collapsed tree shown in Figure 6A.
